# Calcium–Collagen Coupling is Vital for Biomineralization Schedule

**DOI:** 10.1002/advs.202100363

**Published:** 2021-05-27

**Authors:** Jinglun Zhang, Yaoting Ji, Shuting Jiang, Miusi Shi, Wenjin Cai, Richard J. Miron, Yufeng Zhang

**Affiliations:** ^1^ State Key Laboratory Breeding Base of Basic Science of Stomatology (Hubei‐MOST) and Key Laboratory of Oral Biomedicine Ministry of Education School and Hospital of Stomatology Wuhan University Wuhan 430079 China; ^2^ Centre for Collaborative Research Nova Southeastern University Cell Therapy Institute Fort Lauderdale FL 33314‐7796 USA; ^3^ Department of Periodontology College of Dental Medicine Nova Southeastern University Fort Lauderdale FL 33314‐7796 USA; ^4^ Department of Periodontics and Oral Surgery University of Ann Arbor Ann Arbor MI 48109 USA

**Keywords:** biomineralization, bones, bone biomaterials, calcium–collagen coupling, collagen

## Abstract

Biomineralization is a chemical reaction that occurs in organisms in which collagen initiates and guides the growth and crystallization of matched apatite minerals. However, there is little known about the demand pattern for calcium salts and collagen needed by biomineralization. In this study, natural bone biomineralization is analyzed, and a novel interplay between calcium concentration and collagen production is observed. Any quantitative change in one of the entities causes a corresponding change in the other. Translocation‐associated membrane protein 2 (TRAM2) is identified as an intermediate factor whose silencing disrupts this relationship and causes poor mineralization. TRAM2 directly interacts with the sarcoplasmic/endoplasmic reticulum calcium ATPase 2b (SERCA2b) and modulates SERCA2b activity to couple calcium enrichment with collagen biosynthesis. Collectively, these findings indicate that osteoblasts can independently and directly regulate the process of biomineralization via this coupling. This knowledge has significant implications for the developmentally inspired design of biomaterials for bone regenerative applications.

## Graphical abstract

This study clarifies the biological demand pattern of osteoblasts for ER Ca^2+^ concentration and collagen biosynthesis and demonstrates that the translocation‐associated membrane protein 2‐sarcoplasmic/endoplasmic reticulum calcium ATPase 2b (TRAM2‐SERCA2b) regulatory complex can mediate the two‐way relationship during biomineralization. The synchronicity of ER morphology, TRAM2 expression patterns, and SERCA2b activity trends reveal the central position of ER during the entire process of biomineralization.

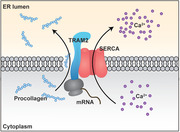

## 1 Introduction

In bone tissue engineering, the promotion of new bone formation similar to that of the original is still the highest goal of bone repair. Numerous bone substitute materials have been designed to regenerate the impaired bone structure. These include bone bioceramics, mesoporous scaffolds, mineralized collagen composites and hydrogels.^[^ ^1^ ^]^ To some extent, these biomaterials can promote the migration, osteogenic differentiation, and osteogenesis of mesenchymal stem cells.^[^ ^1d^ ^]^ However, their direct effects on collagen and calcium phosphates, which are the basic units of bone, are unclear. To design more bionic and efficient materials for clinical applications, knowledge of how normal biomineralization occurs and what osteoblasts need in every phase of new bone formation are required.

Bone formation is a chemical process in which inorganic mineral materials are produced and regulated by organisms.^[^ ^2^ ^]^ Mineralized collagen, the basic building block of hierarchically natural bone, provides mechanical toughness. Hydroxyapatite provides mechanical strength as an inorganic unit.^[^ ^3^ ^]^ To chemically mimic natural bone, scientists have explored the mechanisms of apatite nucleation in vitro and developed collagen mineralization methods.^[^ ^2^ ^]^ Based on these studies, collagen can provide confined nucleation sites, control energy barriers for crystallization, and is responsible for the size and distribution of intrafibrillar apatite crystals in bone, rather than being a passive mineralization template.^[^ ^2^, ^3^, ^4^ ^]^ Although several different nucleation theories have been proposed, the amount of calcium salt and collagen needed in the mineralization system remain unknown.^[^ ^2^, ^4^, ^5^ ^]^ Likewise, the chemical interactions between inorganic materials and collagen are unclear. Other unknowns include whether there is a matched quantitative relationship between collagen and minerals in more physiologically relevant conditions, which is more important. This means it is difficult to completely replicate the biomineralization process in vitro.

On the other hand, biologically, the capability of osteoblasts to form bone can be precisely tuned by several molecular pathways in a context‐dependent manner.^[^ ^6^ ^]^ However, direct modulation of the preparation of these two types of raw materials for biomineralization has not yet been determined. Secretion of collagen and amorphous calcium phosphate (ACP) into the extracellular matrix are two separate processes. After initial processing and transport to the endoplasmic reticulum (ER), tropocollagen molecules are packed into secretory vesicles and released.^[^ ^7^ ^]^ Moreover, calcium ion (Ca^2+^) clusters are generated in the ER and transported into the mitochondria along with phosphate, where they form ACP precursors via mitophagy, and are then exocytosed as matrix vesicles.^[^ ^8^ ^]^ Collagen and inorganic clusters are inextricably linked, either. This imbalance results in very severe conditions, such as osteogenesis imperfecta,^[^ ^9^ ^]^ osteopetrosis,^[^ ^10^ ^]^ and bone fibrous dysplasia.^[^ ^11^ ^]^ Collagen synthesis is a Ca^2+^‐regulated process.^[^ ^12^ ^]^ In the extracellular space, collagen provides the framework and spatial constraints for infiltration, growth, nucleation, and crystallization of ACPs, suggesting that the amount of the mineralizing collagen should match the amount of ACPs.^[^ ^2^, ^3^ ^]^ These observations indicate that the homeostasis and interrelationship between collagen and Ca^2+^ should be directly and finely tuned by cells capable of mineralization. To date, this issue has not been explored.

It is conceivable that the ER may be have a crucial function in this relationship as the basis of most protein synthesis and the main intracellular Ca^2+^ reservoir.^[^ ^13^ ^]^ In the process of osteogenesis, the ER of osteoblasts needs to produce large amounts of type I collagen and absorb large amounts of Ca^2+^.^[^ ^9^, ^14^ ^]^ Our previous study demonstrated that biomineral precursor formation is initiated by transporting calcium and phosphorus clusters from the ER to the mitochondria, suggesting that ER may play a predominant role in biomineralization.^[^ ^8a^ ^]^ Moreover, once the temporary storage and export of these raw materials in the ER are disturbed, it will cause ER stress and even cell apoptosis, leading to failure of bone formation or regeneration.^[^ ^6^, ^14^ ^]^ Therefore, whether in the normal osteogenesis process or in bone repair, the above processes must be correctly controlled.^[^ ^6^, ^12^, ^13^ ^]^ However, the specific mechanism is not well understood.

Studying tissue development is the best way to achieve bone regeneration and apply it to bioengineering medicine. Here, we analyzed natural osteogenesis to determine the coupling mechanism of ER Ca^2+^ uptake and collagen production in osteoblast, in which translocation‐associated membrane protein 2 (TRAM2) is involved as an important regulating protein through its interaction with sarcoplasmic/ER Ca^2+^‐ATPase 2b (SERCA2b). The results of the present study indicate that functional calcium–collagen coupling is essential for biomineralization schedule and emphasize the significance of biomaterial modification with developmentally directional cues to enhance bone tissue regeneration.

## 2 Results

### 2.1 Correlation between Ca^2+^ Concentration and Collagen Content during Biomineralization

**Figure 1:**
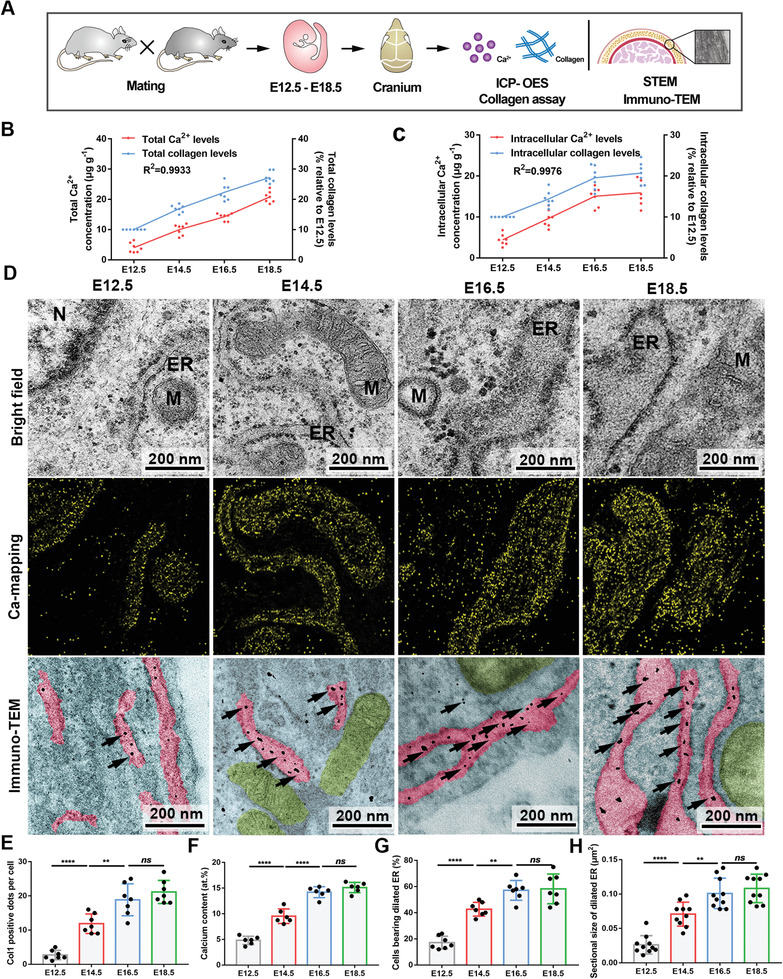
Correlation between Ca^2+^ and Col1 during mineralization in vivo. A) Schematic illustration of the experimental design. Embryonic cranial bones collected at different time points were analyzed by ICP‐OES, STEM and immune‐TEM to determine relationship between the Ca^2+^ and collagen contents during development of cranium. B) Total Ca^2+^ concentrations and collagen levels of whole cranial bones (*N* = 7 replicates; means of each group are connected; ** *P* = 0.0064). C) Intracellular Ca^2+^ concentrations and collagen levels of cranial bones (*N* = 7 replicates; means of each group are connected; ** *P* = 0.0012). D) STEM–EDX elemental mapping and unstained immune‐TEM images of cranial bones. Scale bar = 200 nm. M, mitochondria; N, nucleus; ER, endoplasmic reticulum. Unstained sections are pseudo‐colored. Backgrounds are light blue. ERs are pink. Mitochondria are green. Arrows = Col1. E) The comparison of Col1 positive dots per cell. *N* = 7. F) The comparison of elemental compositions (at%) among regions containing ER in the same area of the groups. *N* = 6. G) The comparison of the percentage of cells bearing dilated ER. Dilated ER elements are defined as ribosome‐studded organelles with expanded lumina (>0.05 µm^2^ in cross‐sectional area). *N* = 7. H) The comparison of sectional size of dilated ER. *N* = 10. E–H) Data represent the mean with standard deviation. ** *P* < 0.005, **** *P*<0.0001, *ns*, no significant difference. All of the experiments are performed at least three times. Unless otherwise stated, data presented in all figures are the mean standard deviation with one‐way ANOVA with Tukey's post‐test.

We hypothesized that the collagen scaffolds and apatite fillers needed for bone tissue formation should accumulate in a balanced pattern during this process. To test this hypothesis (**Figure** **1**A), we investigated the Ca^2+^ concentration and collagen production at several time points during embryonic cranium development as its one‐fold ossification pattern is conducive to analysis. Ca^2+^ and collagen content gradually increased as development progressed and were correlated (*R* ^2^ > 0.99, Figure 1B). In single‐cell dispersions, the intracellular Ca^2+^ and collagen levels both increased until embryonic day (E) 16.5, and then remained approximately the same (Figure 1C). The Pearson's coefficient value also revealed a correlation, suggesting a quantitative correlation between the levels of Ca^2+^ and collagen.

Mesenchymal condensation and mineralization fronts of the cranial bones first localize supraorbital sutures and occur at the margins of the opposed bones.^[^ ^15^ ^]^ To further examine this process microscopically, we examined uniform cross‐sections of cranial bones obtained from E12.5 to E18.5. As shown in Figure S1A in the Supporting Information, collagen 1 (Col1) expression, the calcein‐stained area, and the colocalization of the two colors became increasingly prominent with development (Figure S1B, Supporting Information), consistent with the results in Figure 1B. Scanning transmission electron microscopy (STEM) elemental mapping and immunogold labeling of Col1 (Figure 1D,H) mirrored the trend shown in Figure 1C. Previously, we had demonstrated that the Ca^2+^ needed to form crystals in bone tissue is mainly from the ER, which is also the site of protein synthesis. Therefore, we observed the ER by TEM. Increasingly dilated ER lumens were found in cranial osteoblasts from E12.5 to E16.5. However, there was no significant difference between E16.5 and E18.5 (Figure 1G,H), reflecting the close relationship of ER morphology to Ca^2+^ and Col1 in the process. These findings were further confirmed in bone mesenchymal stem cell (BMSC) samples after osteogenic induction (OI) in vitro (Figure S2, Supporting Information).

Altogether, these findings indicate that the variable trend of Ca^2+^ concentration is consistent with collagen content during osteogenesis in the cranium and BMSCs, regardless of the total or intracellular levels. The ability of cells to store Ca^2+^ and collagen is limited.

### 2.2 Effects of Ca^2+^ Concentration Changes on Collagen Biosynthesis

**Figure 2:**
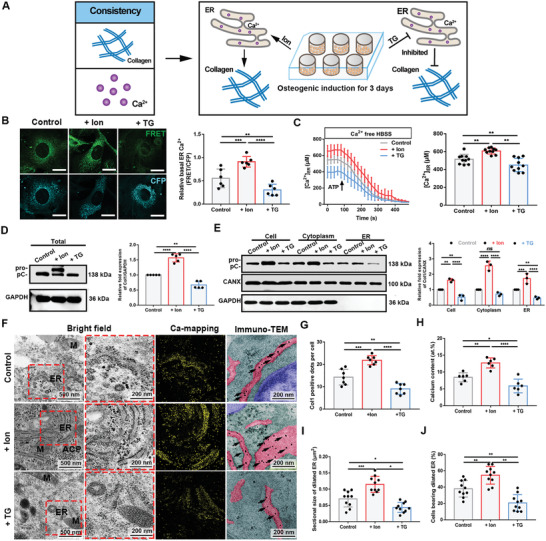
Changes in the Ca^2+^ concentration led to corresponding changes in ER collagen content. A) Schematic illustration. Based on quantitative consistency of Ca^2+^ and collagen, BMSCs underwent osteogenic induction by OM, and then two kinds of treatments was administered for 6 h, followed by determination of Ca^2+^ and collagen changes. Ion, ionomycin; TG, thapsigargin. B) The D1ER fluorescence images. Scale bar = 10 µm. The relative basal FRET ratio (FRET/CFP) of D1ER in three groups. *N* = 6. B) ER Ca^2+^ levels were monitored in control (grey line, *N* = 80), + Ion group (red line, *N* = 65), + TG group (blue line, *N* = 70) after the stimulation of 100 × 10^−6^ m ATP. Error bars represent ± SEM. Histogram shows the average ER Ca^2+^ levels in resting cells. *N* = 10. C) Col1 and GAPDH (loading control) immunoblots of total protein. The three forms of the *α*(I) chain of collagen I: Pro is the unprocessed form with the N‐ and C‐propeptides; pC is collagen with the N‐propeptide cleaved; an *α*1(I) is the fully processed *α*(I) band. Quantification of data. *N* = 5; two‐tailed Student's *t*‐test. D) Col1, GAPDH and CANX (loading control) immunoblots of cell fractions. Quantification of data. *N* = 3; two‐way ANOVA with Dunnett's multiple comparisons test. E) STEM‐EDX elemental mapping and unstained immune‐TEM images. Insets show a high magnification of selected areas. Scale bar = 500 nm and = 200 nm in insets. Unstained sections are pseudo‐colored. Arrows = Col1. F) The comparison of elemental compositions (at%) among regions containing ER. *N* = 6. G) The comparison of Col1 positive dots per cell. *N* = 7. H) The comparison of sectional size of dilated ER. *N* = 10. I) The comparison of the percentage of cells bearing dilated ER. *N* = 10. Cells in this figure are osteogenic induced for 3 days and then treated for 6 h. ** *P* < 0.005, *** *P* < 0.0005, and **** *P* < 0.0001. All of the experiments are performed at least three times.

The foregoing data suggest an interplay between Ca^2+^ enrichment and collagen production in the ER during mineralization. To better investigate this phenomenon, we individually interfered with the content of Ca^2+^ or the content of Col1 in the ER to detect changes in the other variables (**Figure** **2**A). Ionomycin (Ion, 5 × 10^−6^ m), a conventional stimulator of intracellular Ca^2+^ concentrations, and 100 nM thapsigargin (TG), an inhibitor of SERCA that blocks Ca^2+^ transport from the cytosol to the ER, were added to osteogenic‐induced medium (OM) for 6 h after 3 days of OI. Of note, these doses have been reported to be non‐cytotoxic.^[^ ^8a^ ^]^ Additionally, we tested the levels of protein kinase RNA‐like ER kinase (PERK) and phosphorylated PERK (p‐PERK). These treatments were not found to induce improper ER stress (Figure S3A, Supporting Information).

We monitored intracellular Ca^2+^ levels in several experiments. First, the basal cytosolic Ca^2+^ concentrations of the three groups were determined using a well‐recognized fluorescent Ca^2+^ sensor, Fura‐2‐AM.^[^ ^16^ ^]^ (Figure S3B, Supporting Information) The strongest fluorescence signal in the TG group represented the highest basal cytosolic Ca^2+^ levels. Subsequently, Fluo‐4/AM was used to examine cytosolic Ca^2+^ variations. Adenosine 5′‐triphosphate (ATP) can elicit Ca^2+^ release from most intracellular stores.^[^ ^17^ ^]^ Herein, Ion‐induced cells released the most Ca^2+^ into the cytoplasm from Ca^2+^ stores (Figure S3C,D, Supporting Information). These results suggest that most Ca^2+^ are stored in the organelles of the Ion‐group cells.

To directly determine ER Ca^2+^ levels, we used ER‐targeted Cameleon (D1ER), a fluorescence resonance energy transfer (FRET)‐based ER Ca^2+^ indicator.^[^ ^16^, ^17^ ^]^ Ion‐group cells showed weak CFP fluorescence, reflecting increased FRET switching from CFP. The relative basal FRET ratio of the TG‐group cells was only 35% of that of the Ion‐group cells (Figure 2B). The trace diagram of Ion‐treated cells stimulated by ATP also showed a significantly greater reduction in ER Ca^2+^ concentration than that of TG‐group cells (Figure 2C). Another experiment utilized BAPTA‐AM, an intracellular Ca^2+^ chelator, and ethanol, an inducer of ER Ca^2+^ overloading.^[^ ^17^ ^]^ Compared to the dimethyl sulfoxide (DMSO) control, ethanol did not elevate the ER Ca^2+^ concentration due to the reduction in cytosolic Ca^2+^ concentration by BAPTA (Figure S3E, Supporting Information). These data demonstrated that Ion or TG treatment effectively increased or depleted ER Ca^2+^ loading, respectively, with or without OM.

We then investigated the changes in Col1 expression in response to different ER Ca^2+^ concentrations. Western blotting of the total protein (Figure 2D) and cellular fraction proteins (Figure 2E) revealed that Ion‐treated BMSCs produced the largest amounts of Col1. Immunofluorescence analysis also showed similar results (Figure S3F, Supporting Information). TEM images (Figure 2F,J) confirmed that the largest amount of ER Ca^2+^ and most Col1 immunodots were observed in Ion‐treated cells and showed that the frequency of ER enlargement was highest in the Ion group, indicating that ER loading contributed to morphological changes.^[^ ^14^, ^18^ ^]^

To verify the effect of ER Ca^2+^ on mineralization, we added the drugs to the OM after 3 or 7 days of OI. Uniform alizarin red S (ARS) staining was evident on day 14 of OI, demonstrating that Ion treatment resulted in the highest alkaline phosphatase (ALP) activity (Figure S4A,B, Supporting Information) and the most mineral nodules (Figure S4A,C,D, Supporting Information). More importantly, the difference between groups stimulated on day 3 was greater than that between groups stimulated on day 7 (Figure S4E–G, Supporting Information). Furthermore, the transcription levels of several osteogenic markers were detected in the three cell groups. *Col1*, osteocalcin (*Ocn*), alkaline phosphatase (*Alp*), runt‐related transcription factor 2 (*Runx2*), and osterix (*Osx)* were upregulated after Ion treatment (Figure S4H, Supporting Information).

The collective data demonstrate that proper elevations in ER Ca^2+^ concentrations can directly promote Col1 expression and processing in the ER of osteoblasts, while depletion of ER Ca^2+^ has an adverse effect. Ca^2+^ homeostasis during the early stages of mineralization is particularly important.

### 2.3 Effects of Col1 Expression Alteration on Ca^2+^ Concentration

**Figure 3:**
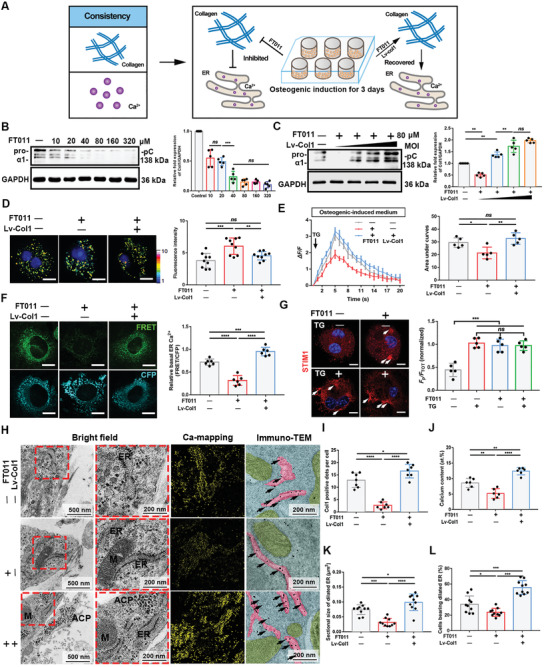
Alteration of the Col1 biosynthesis caused consistent changes in ER Ca^2+^ concentrations. A) Schematic illustration. Based on quantitative consistency of Ca^2+^ and collagen, BMSCs underwent osteogenic induction by OM, and then the collagen expression was inhibited and recovered, followed by determination of Ca^2+^ and collagen changes. Lv‐Col1 indicates lentiviral Col1 plasmids. B,C) The inhibitory effects of FT011 and the retrieval effects of Lv‐Col1 in dose‐dependent manner. *N* = 5. D) Fura‐2‐AM fluorescence changes. Scale bar = 10 µm. Histogram shows the fluorescence intensity (arbitrary units). *N* = 9. E) Cytosolic Ca^2+^ changes tested by Fluo‐4‐AM (Fluo 4) (presented in Δ*F/F*). Error bars represent ± SEM (control group, grey line, *N* = 30; inhibitory group, red line, *N* = 35; retrieval group, blue line, *N* = 30). Bar chart showing the area under the curves (AUC). *N* = 5. F) The D1ER fluorescence images. Scale bar = 10 µm. The relative basal FRET ratio (FRET/CFP) of D1ER. *N* = 6. G) STIM1 localization. Arrowheads = STIM1 puncta. Scale bar = 5 µm. *F*_P_/*F*_TOT_ is calculated as the ratio of fluorescence intensity in the peripheral region (*F*_P_) to the total cell fluorescence (*F*_TOT_). *N* = 5. H) STEM‐EDX elemental mapping and unstained immune‐TEM images. Insets are high magnification of selected areas. Scale bar = 500 nm and = 200 nm in insets. Unstained sections are pseudo‐colored. Arrows = Col1. I,J) The comparison of Col1 positive dots per cell and elemental compositions (at%) among regions containing ER. *N* = 6. K,L) The comparison of sectional size of dilated ER and the percentage of cells bearing dilated ER. *N* = 10. Cells in this figure are osteogenic induced for 3 days and then treated for 2h, with or without transfection. All of the experiments are performed at least three times. *P < 0.05, **P < 0.005, ***P < 0.0005, and ****P < 0.0001, *ns*, no significant difference.

To in turn investigate the effect of collagen expression on Ca^2+^ enrichment in the ER in osteoblasts (**Figure** **3**A), the FT011, an antifibrotic agent, was used to inhibit collagen synthesis.^[^ ^19^ ^]^ The Cell Counting Kit‐8 (CCK‐8) assay demonstrated that the addition of 10 × 10^−6^–80 × 10^−6^ m FT011 did not cause significant cytotoxicity (Figure S5A, Supporting Information). After 3 days of OI, BMSCs were treated with several concentrations of FT011 for 2 h, and the inhibitory effect was detected by western blot. A concentration of 80 × 10^−6^ m was selected for subsequent experiments (Figure 3B). To rescue Col1 expression, lentiviral Col1 plasmids (Lv‐Col1) were transfected into the cells, and Col1 was retrieved in a multiplicity of infection‐dependent manner (Figure 3C). The total collagen levels also confirmed that collagen content was effectively reduced and restored (Figure S5B, Supporting Information). In addition, western blots revealed p‐PERK and PERK expression, indicating that there was no anabatic ER stress under the treatments administered (Figure S5C, Supporting Information).

We proceeded to characterize Ca^2+^ changes in these cells. TG (10 × 10^−6^ m) and Ion (10 × 10^−6^ m) were used to evoke the release of the intracellular Ca^2+^ pool. As shown in Figure 3 D,E, and Figure S5D,E (Supporting Information), the cytosolic Ca^2+^ concentration was increased from the control levels, with the inhibition of collagen expression, indicating the possibility of intracellular Ca^2+^ pool depletion. To directly detect ER Ca^2+^ levels, D1ER was expressed in the three groups of cells. Fluorescence images and the relative ratios (Figure 3F) confirmed the decreased basal ER Ca^2+^ levels in cells treated with FT011 and restoration in cells of the third group. Similarly, examination of ER Ca^2+^ changes caused by ATP or BAPTA stimulation showed decreased ER calcium levels (Figure S5F–H, Supporting Information). Depletion of ER Ca^2+^ can drive the accumulation and redistribution of stromal interaction molecule 1 (STIM1) to the ER‐plasma membrane (ER‐PM) contact zone, promoting compensatory Ca^2+^ influx.^[^ ^16^ ^]^ Immunofluorescence staining typically showed that STIM1 was uniformly distributed (Figure 3G). However, STIM1 clusters were formed after treatment with 10 × 10^−6^ m TG. Even before TG treatment, STIM1 was found to cluster and accumulate in the FT011 group to open the Ca^2+^ influx channels. These data consistently demonstrate that Ca^2+^ levels were lowered in the ER when Col1 expression was inhibited, suggesting that the ER of osteoblasts can modulate Ca^2+^ enrichment based on Col1 content. TEM images also revealed that a reduction in Col1 synthesis led to depletion of ER Ca^2+^ and that Col1 levels were positively correlated with the size of the ER (Figure 3H–L).

We treated BMSCs differently and performed ARS and ALP staining (Figure S6A–D, Supporting Information). The early stage treatment had a greater impact on mineralization than the later stage treatment. Further, stimulation in the later stage had a relatively weak impact (Figure S6D–G, Supporting Information). Determination of the relative mRNA levels further revealed that the expression of key osteogenic markers was inhibited by FT011 (Figure S6H, Supporting Information).

The collective findings indicate that sufficient collagen is particularly required in the early stages of osteogenesis. Col1 expression levels can in turn alter ER Ca^2+^ loading during mineralization, confirming that a balanced two‐way relationship exists between Col1 expression and ER Ca^2+^ levels. Osteoblasts have concordant requirements for Ca^2+^ and collagen to biomineralization. Moreover, the coincidently morphological changes in ER dilation in these experimental groups may indicate that this relationship is related to ER and ER function.

### 2.4 The ER‐Related Proteins Involved in the Bidirectional Relationship

**Figure 4:**
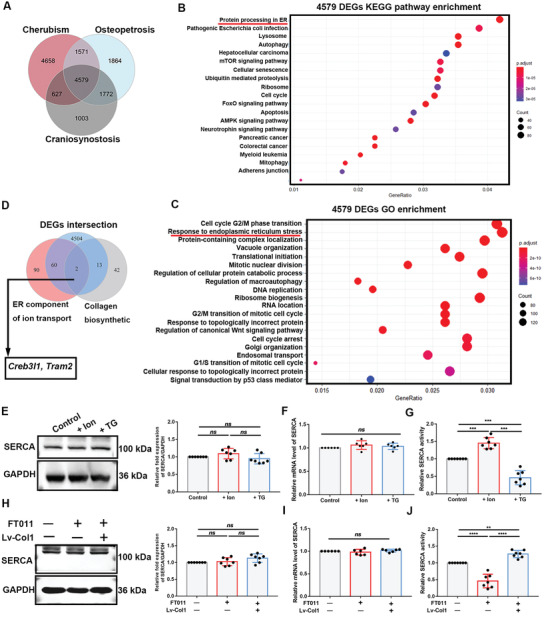
ER‐related proteins were candidates involved in the bidirectional relationship. A) Venn diagram of DEGs. B,C) KEGG pathway enrichment and significant GO terms of differentially expressed genes. DEGs (4593) were analyzed for the KEGG and GO pathway. Bar charts present the significant terms in each category sorted by mean −log^10^ (*P*‐values). Gene Ratio of “Protein processing in ER” is 0.0413 and that of “Response to ER stress” is 0.0315. D) Venn diagram of 2 candidate genes by intersecting differentially expressed genes with ER component of ion transport collection and collagen biosynthetic collection. E,H) SERCA2b and GAPDH (loading control) immunoblots (*N* = 7; normalized fold‐changes were analyzed using two‐tailed Student's *t*‐test comparing the two conditions). F,I) Relative mRNA levels of SERCA2b. *N* = 5; two‐tailed Student's *t*‐test. G,J) Relative SERCA2b activity. *N* = 7; two‐tailed Student's *t*‐test. All of the experiments are performed at least three times. *P < 0.05, **P < 0.005, ***P < 0.0005, and ****P < 0.0001, *ns*, no significant difference.

ER profile changes are the result of fluctuations in Ca^2+^ and protein levels.^[^ ^13^, ^14^, ^18^ ^]^ However, ER is involved in many cellular physiological activities. To determine the type of pathway involved in the balance between Ca^2+^ and collagen during biomineralization, bioinformatics analysis was performed. Databases were selected for the following three bone developmental diseases: cherubism,^[^ ^11^ ^]^ osteopetrosis,^[^ ^10^ ^]^ and craniosynostosis.^[^ ^15b^ ^]^ Cherubism is characterized by excessive replacement of normal bone matrix with fibrous tissue, virtually unrestrained collagen deposition and insufficient calcium salt deposition. Osteopetrosis features bone that is extremely dense but brittle due to excessive mineral deposition and loss of mechanical elasticity conferred by collagen. Craniosynostosis manifests as an untimely accumulation of collagen and inorganic clusters in skull sutures. All three disease are bone developmental disorders associated with an imbalance in the demand for Ca^2+^ and collagen. Venn diagram analysis revealed 4579 common differentially expressed genes (DEGs) among the three databases (**Figure** **4**A). Kyoto Encyclopedia of Genes and Genomes (KEGG) enrichment and Gene Ontology (GO) analysis on these DEGs showed that protein processing in the ER was the top pathway and that genes related to ER stress and protein‐containing complex localization were highly ranked (Figure 4B,C), in addition to genes associated with cell cycle. Thus, ER function is involved in some bone developmental diseases, Ca^2+^ and collagen homeostasis.

SERCA2b is a main active Ca^2+^‐gated influx channel in the ER membrane. SERCA2b has attracted attention as it can maintain ER Ca^2+^ homeostasis and has been widely studied in the context of ER protein processing and Ca^2+^‐independent reactions.^[^ ^12^, ^13^, ^14^, ^16^ ^]^ We intersected the DEGs with ontologies, namely ER Ca^2+^ homeostasis and collagen synthesis genes. Two overlapping genes were eventually obtained (Figure 4B). However, no SERCA2b‐encoding gene were detected. As TG is a direct inhibitor of SERCA2b, we tested SERCA2b in the experimental groups mentioned in Figures 2 and 3. Although no statistically significant differences in SERCA2b expression were observed among the groups, SERCA2b activity was obviously attenuated in TG‐ and FT011‐group cells; however, it was promoted in the Ion‐ and Col1‐retrieval group cells (Figure 4E–J). And *Serca2b* was also involved in ER related significant pathways appeared in KEGG and GO pathways. These data suggest that SERCA2b activity is involved in the ER function of osteoblasts and the relationship between ER Col1 and Ca^2+^.

The foregoing findings suggest that the protein bridge between Ca^2+^ and collagen homeostasis should be associated with ER function and be simultaneously able to modulate SERCA2b activity. To determine which of these two genes was the candidate, the genes were studied separately (Figure S7A–C, Supporting Information). However, the irregular expression observed in the heat maps prompted us to conduct a literature search and further experiments. Cyclic adenosine monophosphate (AMP) responsive element binding protein 3‐like 1 *(Creb3l1*) encodes old astrocyte specifically induced substance (OASIS) that can activate *Col1a1* transcription.^[^ ^14a^ ^]^ OASIS deficiency can lead to severe osteopenia due to a decrease in Col1 in the bone matrix.^[^ ^20^ ^]^ OASIS also acts as a transducer and is induced in response to ER stress.^[^ ^21^ ^]^ However, we found that osteoblasts did not express much more OASIS after Ion treatment (Figure S7D,E, Supporting Information), which might be due to unchanged ER stress levels indicated by p‐PERK expression (Figure S3A, Supporting Information). Moreover, the similar OASIS expression between the control group and the Ion‐group could not explain the promotion of Col1 in the Ion‐group. Col1 was also reduced in the last group, which did not restore Col1 biosynthesis to the normal expression level. TRAM2, a component of the translocon at the ER membrane, is a gated channel that controls the insertion of nascent secretory proteins from the cytoplasm to the ER lumen.^[^ ^22^ ^]^ A previous study proposed that TRAM2 is critical for the biosynthesis of type I collagen in hepatic stellate cells.^[^ ^23^ ^]^ TRAM2 can interact with SERCA2b,^[^ ^23^ ^]^ indicating that it may also control the amount of Ca^2+^ entering the ER lumen via this binding relationship. We observed that treatment with TG and FT011 reduced TRAM2 levels. Ion upregulated the expression of TRAM2 (Figure S7F,G, Supporting Information), coincident with the trend observed in Figures 2 and 3.

Collectively, the data indicate that TRAM2 may be involved in several types of bone developmental diseases and might be capable of affecting SERCA2b in osteoblasts during mineralization and coupling the ER Ca^2+^ concentration with collagen content.

### 2.5 Disruption of the Connection by TRAM2 Knockdown

We investigated the effect of TRAM2 on SERCA2b expression. Briefly, two lentiviral vectors incorporating TRAM2‐targeted small hairpin RNA (Lv‐shTRAM2#1 or #2) were used to knockdown TRAM2 expression in BMSCs. After determining the knockdown efficiency (Figure S8A,B, Supporting Information), SERCA2b activity was found to decrease due to impaired TRAM2 expression. However, SERCA2b protein levels did not change (Figure S8C,D, Supporting Information).

**Figure 5:**
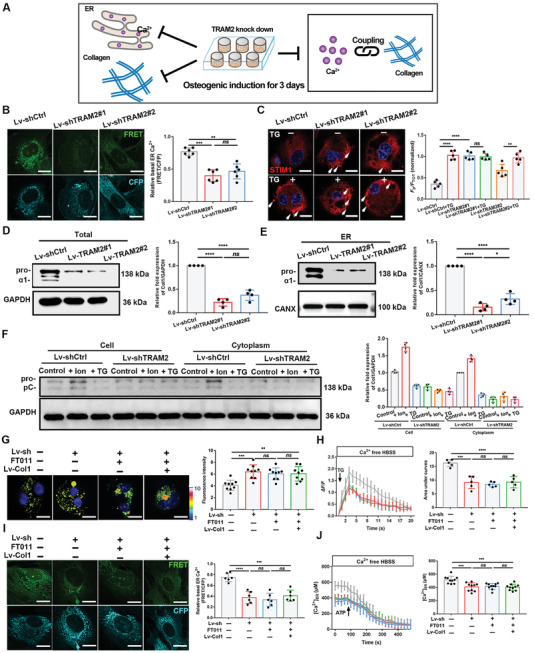
Knockdown of TRAM2 disrupted the connection between Ca^2+^ and Col1. A) Schematic illustration. After bioinformatics analysis and preliminary screening, TRAM2‐knockdown BMSCs underwent osteogenic induction by OM, finding the inhibition and depletion of Ca^2+^ and collagen, with the coupling disrupted. B) The D1ER fluorescence images. Scale bar = 10 µm. The relative basal FRET ratio (FRET/CFP) of D1ER. *N* = 6. C) STIM1 localization. Arrowheads = STIM1 puncta. Scale bar = 10 µm. *F*_P_/*F*_TOT_ calculation is performed as mentioned above. *N* = 5; one‐way ANOVA with Dunnett's multiple comparisons test. D‐E) Col1 and GAPDH (loading control) or CANX (loading control for ER protein) immunoblots of total and ER proteins. *N* = 4; two‐tailed Student's *t*‐test. F) Col1 and GAPDH (loading control) immunoblots of cell (without extracellular matrix) and cytoplasm proteins. *N* = 4. G) Fura‐2‐AM fluorescence changes. Scale bar = 10 µm. Histogram shows the fluorescence intensity (arbitrary units). *N* = 9. H) Cytosolic Ca^2+^ changes tested by Fluo4 (Δ*F/F*) treated in the absence of Ca^2+^. Error bars represent ± SEM (control group, grey line, *N* = 40; knockdown group, red line, *N* = 35; + FT011 group, blue line, *N* = 40; retrieval group, green line, *N* = 45). Bar chart showing the area under the curves (AUC). *N* = 5. I) The D1ER fluorescence images. Scale bar = 10 µm. The relative basal FRET ratio (FRET/CFP) of D1ER. *N* = 6. J) ER Ca^2+^ levels were monitored by D1ER in control group, *N* = 40; knockdown group, *N* = 35; + FT011 group, *N* = 40; retrieval group, *N* = 45, after the stimulation of 100 × 10^−6^ m ATP. Error bars represent ± SEM. Histogram shows the average ER Ca^2+^ levels in resting cells in each group. *N* = 10. Lv‐sh indicates TRAM2 knockdown cells. All of the experiments are performed at least three times. *P < 0.05, **P < 0.005, ***P < 0.0005, and ****P < 0.0001, *ns*, no significant difference.

Such findings indicate that TRAM2 could affect ER Ca^2+^ enrichment. The basal cytosolic Ca^2+^ levels and changes were first measured using Fura‐2 fluorescence reporter and Fluo‐4/AM (Figure S8E–G, Supporting Information). The increase in cytosolic Ca^2+^ in the knockdown groups indicated that the transport of Ca^2+^ from the cytoplasm to the ER was inhibited. The data of fluorescence intensity and ATP‐evoked Ca^2+^ change trace diagram of D1ER expressing cells directly indicated that the ER Ca^2+^ concentration was significantly reduced in TRAM2‐knockdown cells (**Figure** **5**B; Figure S8H, Supporting Information). In addition, the knockdown cells exhibited redistribution of STIM1 puncta to the peripheral PM region in the absence of TG treatment (Figure 5C), confirming the depletion of ER Ca^2+^. Furthermore, Col1 protein expression levels in TRAM2‐knockdown cells were reduced after 3 days of OI in both total cell lysates and ER protein fractions (Figure 5D,E). Hence, TRAM2 can separately influence ER Ca^2+^ oscillation and Col1 production.

As TRAM2 impairment led to decreases in ER Ca^2+^ levels and collagen expression, we explored whether TRAM2 knockdown could terminate the two‐way relationship. Cells transfected with Lv‐shTRAM2 #1 (hereafter referred to as shTRAM2) were treated with Ion or TG. Western blot and confocal microscopy observations (Figure S9A,B, Supporting Information; Figure 5F) demonstrated the significant change of Col1 levels in the Lv‐shCtrl (empty lentiviral vector) group after treatment with Ion or TG, while the expression remained unchanged after stimulation in the Lv‐shTRAM2 group. In addition, TEM images and elemental mapping data revealed that the ER was slender in the experimental group cells, with a lower ER Ca^2+^ concentration and similarly lower collagen expression in these cells than control cells (Figure S9C,F, Supporting Information). The osteogenic ability of knockdown cells did not change significantly after treatment with Ion and TG, as determined by ARS and ALP staining assays (Figure S9D,E, Supporting Information). These results were indicative of the suppressed coupling of Ca^2+^ with Col1 after TRAM2 silencing.

After TRAM2 knockdown, Col1 production in BMSCs was further reduced by FT011 and was restored by Lv‐Col1 (Figure S10A, Supporting Information). Recording of the Ca^2+^ changes revealed that cytosolic Ca^2+^ levels were similarly elevated among the three experimental groups, suggesting that the indiscriminate depletion of intracellular Ca^2+^ pools and inhibition of collagen expression could not further reduce intracellular Ca^2+^ storage after TRAM2 was silenced in cells (Figure 5G,H; Figure S10B,C, Supporting Information). Moreover, FRET data verified that the ER Ca^2+^ concentrations in shTRAM2‐transfected cells were similar to those in cells of other experimental groups (Figure 5I,J). Mineralization induction tests illustrated that inhibition or recovery of collagen expression on the basis of TRAM2 knockdown did not influence mineralization (Figure S10D–F, Supporting Information).

Collectively, these data indicate that TRAM2 is a critical molecular link between ER Ca^2+^ enrichment and collagen content, whose knockdown can disrupt the coupling of ER Ca^2+^ and collagen. TRAM2 might exhibit its function by affecting SERCA2b activity.

### 2.6 TRAM2‐SERCA2b Binding Complex in Biomineralization

**Figure 6:**
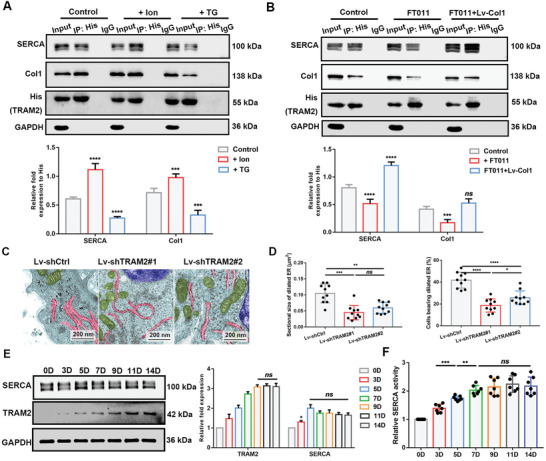
TRAM2 coupled ER Ca^2+^ and collagen by directly binding to SERCA2b and collagen, which is critical to biomineralization. A,B) Using His antibody to pull down, endogenous SERCA2b and Col1 coimmunoprecipitate with His‐tagged TRAM2. *N* = 4; two‐way ANOVA with Tukey's post‐test. C) TEM images. Backgrounds are light blue. ERs are pink. Mitochondria are green. Nuclei are navy blue. Scale bar = 200 nm. D) The comparison of the percentage of cells bearing dilated ER and the sectional size of dilated ER. *N* = 10. E) SERCA2b, TRAM2, and GAPDH (loading control) immunoblots of total proteins. *N* = 4; two‐way ANOVA with Tukey's post‐test. F) Relative SERCA2b activity. *N* = 7; two‐tailed Student's *t*‐test. All of the experiments are performed at least three times. *P < 0.05, **P < 0.005, ***P < 0.0005, and ****P < 0.0001, *ns*, no significant difference.

TRAM2 interacts with SERCA2b and collagen.^[^ ^23^ ^]^ We hypothesized that TRAM2 controls the entry of Ca^2+^ and procollagen into the ER by directly binding to SERCA2b and collagen. To explore the role of TRAM2 in regulating SERCA2b in the bidirectional relationship between Ca^2+^ and Col1, coimmunoprecipitation (co‐IP) was performed using several treatments (**Figure** **6**A,B). Binding was obviously weakened in the TG and FT011 groups but was strengthened in the Ion and rescue groups. SERCA2b activity did not change whether TRAM2 was knocked down or whether the cells were treated with Ion, TG, FT011 or Lv‐Col1 (Figure S11A, Supporting Information). Accordingly, TRAM2 coupled ER Ca^2+^ and collagen by directly binding to SERCA2b and collagen and interfering with SERCA2b activity.

As SERCA2b activity is related to ER function, to determine the effect of TRAM2 on the ER, we characterized the ER profiles in BMSCs after TRAM2 knockdown. After 3 days of OI, TEM images showed that the ER lumen was narrowed in TRAM2‐knockdown BMSCs, indicating a decrease in ER content (Figure 6C). Accordingly, ARS and ALP staining demonstrated that the TRAM2 knockdown group exhibited reduced ALP activity and reduced formation of calcium nodules (Figure S11B–E, Supporting Information). Analysis of the mRNA levels at different time points also showed slowing of osteogenesis due to a lack of TRAM2 (Figure S11F,G, Supporting Information). Therefore, it was also confirmed that TRAM2 was involved in ER function and biomineralization.

To further determine the importance of TRAM2‐SERCA2b binding to Ca^2+^ and Col1 homeostasis, protein samples from the entire mineralization process of BMSCs were collected for western blot analysis. The expression of TRAM2 gradually increased during the first 9 days of induction, and then remained stable. SERCA2b expression was steadier than that of TRAM2 (Figure 6E). However, relative SERCA2b activity first increased and then was maintained without a significant difference after 7 days of OI (Figure 6F). Western blot examination of cranial bone protein samples also revealed a similar trend (Figure S11H,I, Supporting Information). Taken together with the results shown in Figure 1 and Figure S2 (Supporting Information), these findings indicate that normal ER function is regulated by TRAM2 and SERCA2b activity during osteogenesis.

## 3 Discussion

To achieve adequate tissue regeneration or to construct a biomimetic structure, knowledge of the development and formation in the natural state is essential.^[^ ^24^ ^]^ Improved design of bone grafts in tissue engineering requires a better understanding of normal bone biomineralization. Bone development is a process of continuous deposition of Ca^2+^ salts, accompanied by increased collagen mineralization.^[^ ^9^, ^14^ ^]^ The molecular pathways of ossification have been widely studied.^[^ ^6a,c^ ^]^ However, regardless of which mechanism is utilized to promote osteogenesis and recover bone defects, the ultimate goal is to increase Ca^2+^ salt deposition and collagen mineralization, the basic units of bone. Hence, the preparation of raw materials for bone formation is the principal process to be optimized. Loss of ER Ca^2+^ ions can negatively affect collagen production in various types of cells.^[^ ^9^, ^12^, ^18^ ^]^ However, whether Ca^2+^ concentrations and collagen expression can affect each other in osteoblasts, which is crucial for bone formation and regeneration, remains unknown.

The results of the present study reveal that Ca^2+^ enrichment and collagen production can change consistently, indicating that osteoblasts can adjust mineralized raw material preparation according to environmental changes and maximize their use. Moreover, in developing organisms, stem cells can actively regulate developmental processes according to their status.^[^ ^25^ ^]^ Such finding suggests that attention should be paid to the direct effects of research targets or bone‐promoting therapies on Ca^2+^ and collagen when conducting future research on bone.

ER is the main cellular component involved in Ca^2+^ and protein expression.^[^ ^12^, ^13^ ^]^ Consistently, in the bioinformatics analysis of the three bone developmental diseases performed herein, they were also found to be related to ER protein processing. ER dilation and ER Ca^2+^ and Col1 production during biomineralization were also correlated, with levels that increased and then stabilized. Our data further confirm that the ability of the ER to internalize Ca^2+^ and assemble collagen fibrils is finite and interrelated during bone formation. ER dilation is an ultrastructural characteristic that is indicative of Ca^2+^ enrichment and protein processing.^[^ ^14^, ^26^ ^]^ Functional osteoblasts are highly efficient in bone matrix production and are characterized by stacked rough ER layers filled with procollagen.^[^ ^18^, ^20^ ^]^ Active osteoblasts normally appear to moderate ER stress to stimulate the expression of downstream genes that are important for bone development. However, osteoblasts must control ER stress within an acceptable range.^[^ ^14^, ^20^ ^]^ Disturbances in the normal functions of the ER, such as excessive misfolding of proteins and perturbations in Ca^2+^ flux, lead to a negative cell stress response.^[^ ^13^, ^14^, ^26^ ^]^ In the context of osteogenesis, proper regulation of ER conditions is especially important because Ca^2+^ and collagen proteins are the main elements of mineralization. It is reasonable to speculate that there are protective mechanisms in osteoblasts. Nevertheless, many details of this process remain unclear. SERCA2b pumps cytosolic Ca^2+^ into the ER lumen and maintains ER Ca^2+^ homeostasis. SERCA2b has previously been found to be linked to collagen expression through its binding to TRAM2.^[^ ^23^ ^]^ Based on the findings of the present study, we propose that TRAM2‐regulated SERCA2b activity is coincident with ER morphology. TRAM2‐SERCA2b complex activity can be considered as one of the pathways whereby the ER maintains its state during osteogenesis. Our results strongly suggest that the complex functions in a cell‐autonomous manner to preserve osteoblast function. In addition, SERCA2b activity might be involved in various physiological processes and could be affected by more Ca^2+^‐related transmembrane proteins, which is worth further exploration.^[^ ^27^ ^]^ TRAM2 could be related to ER stress. Few studies have addressed the physiological functions of TRAM2, especially in bone tissue. Further studies are required to fully understand the molecular mechanisms of TRAM2 in the prevention of excessive ER stress.

Concerning bone bioengineering, the implanted bone substitute material has various effects on the surrounding bone‐forming cells. For example, MSCs can take up the factors released by the biomaterials, causing a cascade of downstream genes that contribute to bone formation.^[^ ^1^, ^28^ ^]^ However, these current bone tissue engineering strategies always ignore the mineralization demand of osteoblasts, and blindly promote the recovery of bone defects as much as possible.^[^ ^29^ ^]^ Although there are more and more types of bone replacement materials with the development of biomaterial science, they have not greatly shortened the time and cost of bone repair. This is partly due to the lack of understanding of the demand pattern of osteoblast mineralization, which cannot meet the requirements of biomimetic mineralization. Based on findings of our study, there is no need for bone materials to promote both Ca^2+^ uptake and collagen expression of osteoblasts. For those biomaterials that can degrade and release beneficial factors, it is necessary to ensure that releasing process is completed in the early stage of mineralization. Moreover, the mineralization capability of osteoblasts is limited and restricted by the ER. Under the complex physiological environment of bone regeneration, it is quite easy to cause the ER stress of local cells. Therefore, it is important to determine whether grafts induce overwhelming ER stress. It can be inferred that if the materials are functionalized by ER protective agents, such as phenyl butyric acid,^[^ ^30^ ^]^ or by the TRAM2 agonist, they can better enable osteoblasts to fabricate the most conducive components and quicken the bone repair, which should be further assessed.^[^ ^24^ ^]^ The demand pattern of osteoblasts for Ca^2+^ and collagen is also worthy of an in‐depth study and use in bone repair treatment. Therefore, the information obtained from this study may contribute to the better design of bone grafts and high potency of bone regeneration.

Overall, this study revealed the biological demand patterns for Ca^2+^ and collagen during biomineralization. The findings reveal a central physiological function and the active control of the ER in controlling the coupling via TRAM2‐SERCA2b binding in response to OI. The stabilized coupling of Ca^2+^ and collagen is required for new bone formation. Such findings enhance current understanding of biomineralization and will inform deeper explorations of the detailed mechanism of bone regeneration and pathological mineralization.

## 4 Conclusion

**Figure 7:**
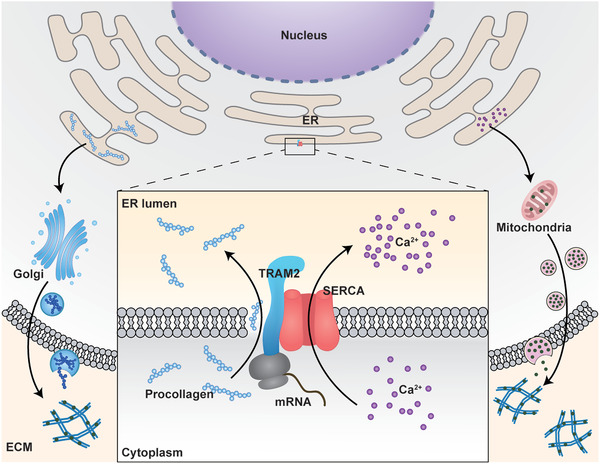
Schematic representation of the calcium–collagen coupling. TRAM2 can bind with naïve collagen protein and maintain the activity of SERCA2b by binding to it, which enables control of the translocation of Col1 and the transport of Ca^2+^ into the ER. Then, Ca^2+^ clusters and collagen are transported into the mitochondria and Golgi apparatus, respectively, followed by exocytosis, to be involved in bone matrix mineralization.

This study clarifies the biological demand pattern of osteoblasts for ER Ca^2+^ concentration and collagen biosynthesis and demonstrates that the TRAM2‐SERCA2b regulatory complex can mediate the two‐way relationship during biomineralization. Once such regulation is lost, osteoblasts do not independently control the reserves of Ca^2+^ and collagen to adjust the schedule of mineralization. Mechanistically, TRAM2 combines with naïve collagen protein and maintains the activity of SERCA2b by binding to it, which enables the control of Col1 translocation and Ca^2+^ transport to the ER (**Figure** **7**). TRAM2 knockdown directly leads to ER constriction and blocks mineralization. In addition, the synchronicity of ER morphology, TRAM2 expression patterns and SERCA2b activity trends revealed the central position of ER during the initiation as well as the entire process of biomineralization. Therefore, the amount of collagen and calcium salts should be matched to build an appropriate mineralization structure and enable the best use of materials. The requirements for biomineralization for the calcium and collagen are concordant. The ER function and the balance above should be focused to improve the regenerative effects of bone substitute materials.

## 5 Experimental Section

### Mice and Sample Collection

All animal experiments were conducted in compliance with the policies of the Ethics Committee for Animal Research, Wuhan University, China (ethical approval number 2017/69). Eight‐week‐old female and ten‐week‐old male C57BL/6 mice to mate and five‐week‐old female C57BL/6 mice for collecting BMSCs were purchased from Vital River (China). The observed plug date is denoted as E0.5. Before harvesting the embryos, pregnant female mice were sacrificed by an overdose of anesthetic.^[^ ^8^, ^31^ ^]^ More details are provided in the Supporting Information.

### Cell Culture, Transfection, and ALP and ARS Staining

BMSCs were collected from the femurs and tibias of female 5‐week‐old C57BL/6 mice by digesting bone chips with collagenase type II (1 mg mL^−1^, 17 101 015, Gibco, Life Technologies Corporation) as described previously.^[^ ^32^ ^]^ A mouse TRAM2‐specific shRNA lentivirus vector (pLVX, GeneChem, China) was used for the knockdown of TRAM2 and designated as Lv‐shTRAM2#1 or #2. The sequences are listed in Table S1 in the Supporting Information. More details are provided in the Supporting Information.

### Treatments

To properly regulate the Ca^2+^ concentration in the ER, 5 × 10^−6^ m Ion (S1672, Beyotime, China) and 100 nM TG (T9033, Sigma‐Aldrich, USA) were added to the OM 3 days after OI based on our previous results.^[^ ^8a^ ^]^ To inhibit the expression of type I collagen, BMSCs were transfected with empty lentiviral vector and treated with the collagen inhibitor, FT011 (HY‐100495, MedChemExpress, USA) for 2 h.^[^ ^19^ ^]^ To stimulate STIM1 redistribution, 10 × 10^−6^ m TG was used for 10 min. To chelate cytosolic Ca^2+^, 40 × 10^−6^ m 1,2‐bis(o‐aminophenoxy)ethane‐*N*,*N*,*N*′,*N*′‐tetraacetic acid (BAPTA, HY‐100168, MedChemExpress, USA) or an equivalent amount of DMSO was added to the OM solution. Additional details were provided in the Supporting Information.

### Quantification of Ca^2+^ and Collagen

The total and intracellular Ca^2+^ contents of mineralized cells and skull samples were determined by inductively coupled plasma optical emission spectrometry (ICP‐OES). Quantification of total and intracellular collagen was performed using a Sircol soluble collagen assay kit (Biocolor, UK), according to the manufacturer's protocol and the methods used in previous studies.^[^ ^6^, ^14^ ^]^ Additional details are provided in the Supporting Information.

### Histological Analysis of Paraffin Specimens

Undecalcified embryonic calvaria were collected (due to the relatively low calcium deposition in mature bone) at several time points, gradient‐dehydrated and embedded in paraffin. Dehydration was performed with 30%, 50%, 70%, 80%, 90%, and 95% ethanol for 1 hour at each gradient. Staining of 5‐µm paraffin sections with hematoxylin‐eosin (MXB Biotechnologies, China) was performed according to standard protocols. Additional details are provided in the Supporting Information.

### Ultrastructural Examination

Cells and calvaria were harvested, fixed, dehydrated, permeabilized, and embedded in epoxy resin as previously reported.^[^ ^8^, ^31^ ^]^ More details are provided in the Supporting Information.

### Relative SERCA2b Activity Measurement

To purify the microsomes enriched in the ER membranes, treated cells cultured in 15‐cm dishes were digested and suspended on ice in homogenization buffer (250 × 10^−3^ m sucrose, 5 × 10^−3^ m HEPES buffer, 1 × 10^−3^ m phenylmethylsulfonyl fluoride [PMSF] PMSF).^[^ ^16^, ^27^, ^33^ ^]^ More details are provided in the Supporting Information.

### RNA Extraction and RT‐qPCR

Total RNA was extracted with TRIzol reagent (Thermo Fisher Scientific, USA) from BMSCs cells. After isolation with chloroform and isopropyl alcohol and purification with ethanol, complementary DNA was synthesized. RT‐qPCR was performed using a Prime‐Script RT‐PCR Kit (TaKaRa Bio, Japan). The primer sequences are listed in Table S2 in the Supporting Information. Glyceraldehyde‐3‐phosphate dehydrogenase (*Gapdh*) was used as the reference gene. PCR cycle steps are as follows: Stage 1, pre‐degeneration, at 95 °C for 30 seconds; Stage 2, Circular reaction, repeats 40 times, 95 °C for 10 seconds and 60 °C for 30 seconds; Stage 3, Dissolve curve, 95 °C for 15 seconds, 60 °C for 60 seconds and 95 °C for 15 seconds.

### Protein Collection and Western Blotting

To extract total proteins, cells were scraped and lysed using RIPA lysis buffer containing 1 × 10^−3^ m PMSF. The anti‐collagen 1 antibody used recognizes the procollagen alpha1(I) chain with the N‐ and C‐propeptides, the pC‐propeptide chain (pC‐alpha1(I)), and the alpha1(I) chain with both the N‐ and C‐propeptides cleaved.^[^ ^12a^ ^]^ To separate cells and extracellular matrix fractions, a method using deoxycholate was modified from a previous study.^[^ ^34^ ^]^ To isolate the ER fractions, 10^9^ cells were digested from 15‐cm dishes, washed with IB_cells_‐2 buffer (30 × 10^−3^ m Tris‐HCl, pH = 7.4, 225 × 10^−3^ m mannitol, and 75 × 10^−3^ m sucrose), and pelleted at 600 × *g* for 5 min at 4 °C, as previously described.^[^ ^35^ ^]^ Additional details are provided in the Supporting Information.

### Co‐IP

For co‐IP, cells were transfected with a TRAM2‐target lentiviral vector containing a His tag at the N‐terminus (GeneChem, China). Additional details are provided in the Supporting Information.

### Confocal Laser Scanning Microscopy

To visualize fluorescent Ca^2+^, ER, Col1, and STIM1, cells were treated, and images acquired using an InSIGHT Plus‐IQ microscope, (Meridian, USA). Additional details are provided in the Supporting Information.

### Cytosolic ([Ca^2+^]_cyto_) and ER ([Ca^2+^]_ER_) Ca^2+^ Measurements

To visualize cytosolic Ca^2+^, the Fura‐2‐AM fluorescent Ca^2+^ sensor (S1052, Beyotime, China) was used.^[^ ^36^ ^]^ To evaluate Ca^2+^ changes in the cytoplasm after treatment, the Fluo‐4/AM fluorescent Ca^2+^ sensor (S1060, Beyotime, China) was used according to previous studies.^[^ ^16^, ^17^ ^]^ [Ca^2+^]_ER_ detection with D1ER (pcDNA‐D1ER was a gift from Amy Palmer and Roger Tsien (Addgene plasmid #36 325; http://n2t.net/addgene:36325; RRID:Addgene_36 325) was performed using an Olympus FV1000 confocal microscope as previously described.^[^ ^17^, ^37^ ^]^ To chelate cytosolic Ca^2+^, 40 × 10^−6^ m BAPTA or an equivalent amount of DMSO was added to the solution. The OM was refreshed with OM containing 8% ethanol to stimulate ER Ca^2+^ inflow. The medium was then replaced with 100 × 10^−6^ m ATP·2Na·3H2O or 10 × 10^−6^ m TG in Ca^2+^‐free Hank's Balanced Salt Solution. Data analysis was performed using GraphPad Prism 7.0 (GraphPad, USA). Additional details are provided in the Supporting Information.

### Bioinformatics Analysis

Series matrix files of GSE146111, GSE55282, and GSE139421 were downloaded from the Gene Expression Omnibus databases (GEO, http://www.ncbi.nlm.nih.gov/geo/). They were based on GPL18480 (Illumina HiSeq 1500), GPL11154 (Illumina HiSeq 2000), and GPL19057 (Illumina NextSeq 500), respectively. The GSE146111 dataset contained tissue samples from four loss‐of‐function mutants, two gain‐of‐function mutants, and two wild‐type controls of hypertrophic chondrocyte descendants. The GSE55282 dataset contained 23 craniosynostosis skull samples and eight normal samples. The GSE139421 dataset contained six biological replicates samples of bone marrow derived from from 6 to 8‐week‐old wild‐type and cherubism mice.

Raw data were converted into an expression matrix using a platform information file. DEG screening between loss‐of‐function mutants and wild‐type control was carried out using the R package, limma (3.6.3).^[^ ^38^ ^]^ The same software with default parameters was used to identify DEGs between the craniosynostosis skull samples and normal samples. The DESeq2 package^[^ ^39^ ^]^ with default parameters was used to normalize the raw counts and identify DEGs in MSC samples between cherubism and wild‐type mice. Log_2_ fold change ≥ ± 1 with an adjusted *p*‐value < 0.05 was set to obtain DEGs.

To analyze the potential biological processes, cellular components, molecular functions, and pathways of the overlapping DEGs, the R package, clusterProfiler (3.12.0),^[^ ^40^ ^]^ with default parameters was used to perform GO analysis and KEGG pathway enrichment analysis. Ontology data of GO: 00 32469 (endoplasmic reticulum calcium ion homeostasis, 2019‐01‐01) and GO:00 32964 (collagen biosynthetic process, 2019‐01‐01) were downloaded from AmiGO^61^. The intersection of the overlapping DEGs and GO:00 30176 (species: *Mus musculus*) and GO:00 32964 (species: *Mus musculus*) was performed using RStudio and the result was visualized using the R package, Venn diagram.^[^ ^41^ ^]^

The heatmaps shown the normalized data of *Creb3l1* and *Tram2* expression in three databases. GSE146111 and GSE55282 are FPKM data types. Data were based on Limma's TMM normalization, plus one, and then calculates the base‐10 logarithm of the values. GSE139421 is a Count datatype. It used the normalization method that comes with DEseq2.

### Statistical Analyses

Data are presented as mean ± SEM or mean with standard deviation as indicated in the figure legends. For comparisons between more than two experimental groups, the statistical significance of normally distributed data was analyzed by one‐way analysis of variance (ANOVA) with Tukey's post‐hoc test (see figure legends). Two‐tailed Student's *t*‐test was used for comparisons to normalize values for the control group. GraphPad Prism v.7 software was used for the statistical analyses. The level of significance is indicated by asterisks. *P* values of less than 0.05 were considered as statistically significant. No statistical method was used to estimate sample sizes. Independent experiments were conducted several times using different sample replicates. BMSCs were randomly allocated to different experimental groups.

## Conflict of Interest

The authors declare no conflict of interest.

## Supporting information

Supporting Information

## Data Availability

Research data are not shared.
