# Supplementary material for: Calcium–Collagen Coupling is Vital for Biomineralization Schedule
Source: Adv Sci (Weinh). 2021 May 27;8(15):2100363. doi: 10.1002/advs.202100363 (PMC8336496; doi:10.1002/advs.202100363)
Supplement: Supplementary file 1 — Supporting Information [file ADVS-8-2100363-s001.pdf]

© 2021 Wiley-VCH GmbH

ADVANCED SCIENCE
Open Access

Supporting Information

for *Adv. Sci.,* DOI: 10.1002/advs.202100363

Calcium-collagen coupling is vital for biomineralization schedule

Jinglun Zhang[a], *Yaoting Ji*[a], *Shuting Jiang*[a], *Miusi Shi*[a], *Wenjin Cai*[a], *Richard J. Miron*[bcd], *Yufeng Zhang*[a]*

Supplementary Information

Calcium-collagen coupling is vital for biomineralization schedule

Jinglun Zhang, Yaoting Ji, Shuting Jiang, Miusi Shi, Wenjin Cai,
*Richard J. Miron, Yufeng Zhang**

*Corresponding author. E-mail: zyf@whu.edu.cn (Y.F.Z.)

This file includes:

Supplementary Materials and Methods
Fig. S1. Histological analysis of cranium collected from different time points.
Fig. S2. Correlation between Ca^{2+} and Col1 during mineralization *in vitro*.
Fig. S3. Characteristics of Ca^{2+} changes after treatments of Ion or TG.
Fig. S4. Effects of ER Ca^{2+} changes on mineralization.
Fig. S5. Characteristics of Ca^{2+} concentrations after collagen inhibition and retrieval.
Fig. S6. Effects of collagen expression changes on mineralization.
Fig. S7. Preliminary determination of TRAM2 as candidate.
Fig. S8. Ca^{2+} characteristics of TRAM2-knockdown cells.
Fig. S9. The useless effects of Ion and TG on TRAM2-knockdown cells.
Fig. S10. The effects of Col1 expression changes on TRAM2-knockdown cells.
Fig. S11. The importance of TRAM2-SERCA2b in osteogenesis.
Table S1. The shRNA sequences used in study.
Table S2. The primer sequences used in study.

Supplementary Materials and Methods

Mice and sample collection: To observe Ca^{2+} deposition in the skull, calcein-AM (16 mg/kg body weight; C001, Dojindo, Japan) was injected intraperitoneally 4 days before sacrifice. To make paraffin sections, the whole calvaria tissues were fixed with 4% paraformaldehyde at 4 °C overnight. Tissues intended for ultrastructural studies were fixed with 2.5% glutaraldehyde in 0.1 M phosphate buffer (PB, pH = 7.4) at 4 °C overnight. Other samples intended for immuno-TEM were quickly fixed with a solution of 2% paraformaldehyde and 0.5% glutaraldehyde in 0.1 M PB (pH = 7.4) at 4 °C overnight. The same area of calvaria with supraorbital cranial sutures were fixed in these fixation fluids. To ensure that the ultrastructure of the same region was observed in different samples, the calvaria were observed at supraorbital sutures, consistent with the methods in previous studies.[1]

Cell culture, transfection, and ALP and ARS staining: BMSCs were collected from the femurs and tibias of female 5-week-old C57BL/6 mice by digesting bone chips with collagenase type II (1 mg mL^{-1}, 17101015, Gibco, Life Technologies Corporation) as described previously.[2] The cells were cultured in alpha minimum essential medium (αMEM; Gibco, Life Technologies Corporation, USA) containing 20% fetal bovine serum (FBS; Gibco, Life Technologies Corporation, USA). Further purified cells (passage 3-10) were used in subsequent experiments. The osteogenic-inducing medium (OM) was composed of 10% FBS, 10 nM dexamethasone (D4902, Sigma-Aldrich, USA), 10 mM β-glycerophosphate (G9422, Sigma-Aldrich, USA), and 5 mM L-ascorbic acid (A4403, Sigma-Aldrich, USA) in αMEM. The OM of each well was replaced with fresh OM every other day. BMSCs were induced to

differentiating into osteoblasts by 3 days of OI.[2]

A mouse TRAM2-specific shRNA lentivirus vector (pLVX, GeneChem, China) was used for knockdown of the TRAM2 and designated as Lv-shTRAM2#1 or #2. The sequences are listed in Table S1. An empty lentiviral vector only was used as a control (Lv-shCtrl). The cells were transfected with virus particles (35 particles per cell) with the help of 5 μg/mL hexadimethrine bromide (Polybrene, Millipore Sigma, USA).

Mineralized cells for ALP staining were prepared after incubation for 7 days. BMSCs were fixed and treated with an ALP staining kit (C3206, Beyotime, China), after with the enzyme activity was determined (P0321S, Beyotime, China). After incubation with working solution, the absorbance was tested immediately to determine the optical density (OD) of 405 nm. Additionally, total protein was extracted, and the concentration was quantified by the bicinchoninic acid (BCA) method (23250, Thermo Fisher Scientific, USA). ALP activity was normalized and calculated based on the OD405 value per milligram of total protein. After being induced for 14 days, cells in well plates were stained with 0.1% Alizarin red S (A5533, Sigma-Aldrich, USA) solution (pH = 4.6). After pictures were obtained, the Alizarin red was dissolved in 10% cetylpyridinium chloride (C9002, Sigma-Aldrich, USA) at room temperature for 4 h, and the absorbance value (OD value) was examined at 562 nm. In terms of the design of the ARS staining experiments, cells were treated with Ca^{2+} flux regulators after OI for 3 days and 7 days. The treatment time was 6 h. The medium containing drug was then discarded, and fresh OM was added. Moreover, the OM was discarded at 3 or 7 days after OI and the cells were treated with 80 μM FT011 for 2h or transfected with pLVX-Col1 lentiviral vector (multiplicity of infection, MOI = 40). The medium of former group was then

changed with αMEM (containing 10% FBS) to wait for the transfection of latter group. Then the transfection reagents were replaced with OM after transfection completion. The duration of actual osteogenic induction was 14 days.

Treatments: To properly regulate the Ca^{2+} concentration in the ER, 5 μM Ion (S1672, Beyotime, China) and 100 nM TG (T9033, Sigma-Aldrich, USA) were added to the OM 3 days after OI based on our previous results.[2] After 6 h of treatment, cells were collected for subsequent experiments. To inhibit the expression of type I collagen, BMSCs were transfected with empty lentiviral vector and treated with the inhibitor, FT011 (HY-100495, MedChemExpress, USA) for 2 h. The concentrations of FT011 were as follows: 10 μM, 20 μM, 40 μM, 80 μM, 160 μM and 320 μM.[3] For rescue experiments, different virus particles expressing the lentiviral vector pLVX-Collagen1 (MOI: 5, 10, 20 or 40 particles per cell, GeneChem, China) were transfected into target cells and cells were purified by 6 μg mL^{-1} puromycin. After determination of rescue effectiveness, an MOI of 40 was used in the following experiments. When both viruses were transfected, the cells were first transfected with Lv-shTRAM2 and formed into a stable cell strain before transfection with the next kind of virus. To transiently evoke Ca^{2+} flux, 10 μM Ion, 10 μM TG or 100 μM ATP was used in the indicated experiments.[4] To stimulate STIM1 redistribution, 10 μM TG was used for 10 min.[4a] To chelate cytosolic Ca^{2+}, 40 μM 1,2-bis(o-aminophenoxy)ethane-N,N,N′,N′-tetraacetic acid (BAPTA, HY-100168, MedChemExpress, USA) or an equivalent amount of DMSO was added to the OM solution.[4b]

Quantification of Ca^{2+} and collagen: The total and intracellular Ca^{2+} contents of mineralized cells and skull samples were determined by inductively coupled plasma optical emission spectrometry (ICP-OES). BMSCs were cultured in 15-cm dishes, induced by OM and were detached on day 1, 5, 9 and 14. Collected cells and calvaria were washed three times with phosphate buffer solution (PBS). Prior to measurement, the cell suspension or tissues were wash with deionized water and diluted 7.5-fold with 2% concentrated nitric acid (Sigma-Aldrich, USA). To assess intracellular Ca^{2+} levels, cells or calvaria were digested with 0.1% collagenase (1 mg/mL, 17101015, Gibco, Life Technologies Corporation, USA) and 0.2% dispase (17105041, Gibco, Life Technologies Corporation, USA) to remove matrix components, without disrupting cell activity. After the impurities were removed, the cell suspension was centrifuged for subsequent steps of ICP-OES.

Quantification of total and intracellular collagen was performed using a Sircol soluble collagen assay kit (Biocolor, UK) according to the manufacturer's protocol and the methods used in a previous study.[5] Briefly, 10^9 mineralized cells or micro-dissected calvaria were treated with acid pepsin and mixed with Sircol dye. The absorbance was tested at 555 nm and a standard curve was made using rat tail collagen (354236, Corning, USA). To evaluate intracellular collagen production, single cells were first collected as described above. Then, the OD values were measured and analyzed. The data from each group were normalized according to the data of corresponding control group.

CCK-8 assay: The cytotoxicity of FT011 on BMSCs was tested by CCK-8 kit (CK04,

Dojindo, Japan) after 1, 3, 5 and 7 days of culture with different concentrations of FT011 in OM. Cells were seeded at a density of 6×10^3 cells per well in a 96-well plate. At each time point, the medium was discarded and replaced with 110 μL of working solution according to manufacturer's protocol. Then, the cells were reacted for 1 h at 37 °C. The OD values were quantified by measuring the absorption at 450 nm as soon as possible.

Histological analysis of paraffin specimens: For immunofluorescent staining, the 5-μm paraffin sections were incubated with antibodies against Col1 (1:100, A16891, ABclonal, USA). The secondary antibody DyLight 594 goat anti-rabbit IgG (1:500, A23420, Abbkine, USA) was used. The nuclei of cells were stained with 4-6-diamidino-2-phenylindole (DAPI, C1002, Beyotime, China). An olympus DP72 microscope was used to capture calcein (green) and red fluorescence images. The green areas, representing calcium deposits were measured with Image-Pro Plus 6.0 software (National Institutes of Health, U.S.A.). Cells with positive collagen 1 expression were counted relative to the total number of cells. Semi-quantitative analyses were performed by an investigator blinded to the samples.

Ultrastructural examination: Cells and calvaria were harvested, fixed, dehydrated, permeabilized, and embedded in epoxy resin as previously reported.[1b, 6] To measure the Ca^{2+} distribution within the ER, 70-nm sections were observed by 100 kV high-resolution scanning TEM (STEM, HT-7700, Hitachi, Tokyo, Japan) coupled with energy-dispersive X-ray spectroscopy (EDX).[7] To quantify the chemical elements, regions of the same area that contained the ER were selected with an Aztec nanoanalysis system (Oxford Instruments, UK).

The boundary of the ER was identified by the membrane. The average percentages of calcium content were statistically analyzed.

Immuno-TEM was used to identify collagen molecules within ER lumen. Cells were embedded with LR-White resin (Ted Pella, USA). Grids were treated with 1% H_2O_2 in phosphate buffer, neutralized with ammonium chloride, blocked with 5% bovine serum albumin, and incubated with a primary antibody against Col 1 (1:100, A16891, ABclonal, USA) and then conjugated with 1.4 nm nanogold (Nanoprobes, USA). The samples were examined with a JEM-1400 transmission electron microscope (JEOL, Tokyo, Japan) at 120 kV. Equal areas were randomly selected in each figure, and the number of positive dots per cell was calculated.

To describe ER activity, dilated ER elements were defined as ribosome-studded organelles with expanded lumina (with cross-sectional areas of more than 0.05 μm^2). The proportion of cells with a dilated ER and the averaged luminal size of each dilated ER-containing cell were determined, and statistical comparisons were performed.

Relative SERCA2b activity measurement: To purify the microsomes enriched in the ER membranes, treated cells cultured in 15-cm dishes were digested and suspended on ice in homogenization buffer (250 mM sucrose, 5 mM HEPES buffer, 1 mM phenylmethylsulfonyl fluoride [PMSF]).[4a, 8] The mixtures were centrifuged at 1,000 × *g* for 10 min at 4 ℃, and then the supernatants were centrifuged at 12000 × *g* for 15 min at 4 ℃. The new supernatants were centrifuged for 60 min at 100000 × *g* at 4 ℃ (70-Ti rotor, Beckman). The pellets were collected and resuspended in homogenization buffer. The protein concentration was

determined by the BCA method. SERCA activity was measured with a colorimetric ATPase assay kit in accordance with previous research. Isolated microsome fractions were added as 30 μg mL^{-1} of protein into reaction buffer containing 100 mM KCl, 10 mM $MgCl_2$, 20 mM HEPES (pH = 7.0), 10 mM phosphoenolpyruvate, 1 mM EGTA, 15 U mL^{-1} each of pyruvate kinase and lactate dehydrogenase, 0.5 mM NADH, and 2 μM calcimycin (A23187, Sigma, USA). The reactions were performed with or without 5 μM free Ca^{2+} and were started by adding 5 μM ATP. The decrease in absorbance at 340 nm after 10 min of incubation at 37 °C was recorded using a BioTek Epoch Microplate Spectrophotometer. SERCA2b-independent Ca^{2+}-ATPase activity was measured in the presence of the thapsigargin (10 μM). The activity of SERCA2b was calculated as hydrolyzed ATP level (nmol) normalized to the protein content (mg) and reaction time (min) from three independent experiments. The activity of SERCA was normalized by protein content and is expressed relative to the value in the control group.

Protein collection and western blotting: To extract total proteins, cells were scraped and lysed using RIPA lysis buffer containing 1 mM PMSF. Proteins were loaded for electrophoresis by SDS-PAGE and then transferred to a polyvinylidene difluoride membrane. After blocking with 5% nonfat milk for 1 h, the membranes were incubated with primary antibodies at 4 °C overnight and corresponding secondary antibodies for 1 h at room temperature, before visualization with a WesternBright ECL HRP substrate kit (Advansta, USA) and ChemiDoc™ Imaging System (#12003153, Bio-rad, USA). Primary antibodies against the following proteins were used: SERCA2b (1:1000, Abclonal, USA, Catalog No. A1097), TRAM2

(1:10000, Abcam, UK, Catalog No. ab109176), GAPDH (1:5000, ABclonal, USA, Catalog No. AC002), Calnexin (CANX; 1:1000, ABclonal, USA, Catalog No. A15631), Phospho-PERK (1:1000, ABclonal, USA, Catalog No. AP0886), PERK (1:1000, ABclonal, USA, Catalog No. A18196) and Col1 (1:1000, ABclonal, USA, Catalog No. A16891). The anti-collagen 1 antibody used recognizes the procollagen alpha1(I) chain with the N- and C-propeptides, the pC-propeptide chain (pC-alpha1(I)), and the alpha1(I) chain with both the N- and C-propeptides cleaved.[9]

To separate cells and extracellular matrix (ECM) fractions, a method using deoxycholate (DOC) was modified from a previous study.[9b] After treatment, plates were washed with PBS. Cells and ECM were scraped with 300 μL of 4% DOC solution containing 20 mM Tris-HCl (pH = 8.8), 1 mM PMSF and 200 μU DNase. Then, the extracts were pelleted at 17000 × *g* for 30 min at 4 ℃. The supernatant was kept as the DOC-soluble fraction, which included cellular material and components not incorporated into the ECM, while the DOC-insoluble pellets contained the ECM. The former was added to loading buffer for subsequent western blotting analysis.

To isolate the ER fractions, 10^9 cells were digested from 15-cm dishes, washed with IB_{cells}-2 buffer (30 mM Tris-HCl, pH = 7.4, 225 mM mannitol and 75 mM sucrose), and pelleted at 600 × *g* for 5 min at 4 ℃, as previously described.[10] The pellet was resuspended in IB_{cells}-1 buffer (30 mM Tris-HCl, pH = 7.4; 225 mM mannitol; 75 mM sucrose; 1 mM PMSF; and 0.1 mM EGTA) and blended with a Dounce homogenizer at 4 ℃. Then the mixture was centrifuged twice at 600 × *g* for 5 min at 4 ℃ and the supernatants were centrifuged at 7000 × *g* for 15 min at 4 ℃. The supernatants were further centrifuged at 20000 × *g* for 40 min at 4 ℃,

and the resulting supernatants were centrifuged at 100000 × *g* for 90 min at 4 ℃. The pellets were resuspended with IB_{cells}-2 buffer and considered the ER compartment. The lysates were added to loading buffer for subsequent western blotting assay. The quality of ER protein was confirmed with different markers (with calnexin as an ER marker).

To collect calvaria proteins, cranial bones were carefully isolated and micro-dissected, followed by washing with PBS three times and lysed with RIPA lysis buffer containing 1 mM PMSF for 30 min on ice. Subsequently, mixtures were centrifuged and performed BCA assay, then denatured for subsequent western blotting process.

Co-IP: For co-IP, cells were transfected with a TRAM2-target lentiviral vector containing a His tag at the N-terminus (GeneChem, China). After induction by the indicated treatments, the cells were rinsed with PBS and lysed at 4 °C with Nonidet P 40 (NP-40, P0013F, Beyotime, China). Then, the cells were collected in the microcentrifuge tubes and shaken slowly on ice on a shaking table for 30 minutes. The supernatant was obtained after centrifugation, and the protein concentration was measured by the BCA method. A His primary antibody (1:1000, ABclonal, USA, Catalog No. AE003) or negative control IgG was added to the protein. The protein was rotated gently overnight at 4 °C. Forty microliters of protein A/G magnetic beads for IP (B23202, Bimake, USA) were added to proteins and rotated gently for 1 hour at room temperature. Then the mixture was washed using NP-40 buffer, and the protein was resuspended in 50 μL of SDS. After denaturation at 95 °C, the samples were analyzed by western blotting. To compare interaction level among groups, loading volume was adjusted based on His expression level of antibody groups.

Confocal laser scanning microscopy: To visualize fluorescent ER and Col1, cells in different groups were stained with a live cell dye, ER-Tracker Red (1:500, C1041, Beyotime, China), for 30 min at 37 ℃. Then, the cells were fixed in 4% paraformaldehyde for 10 minutes, permeabilized with 0.5% Triton-X-100, blocked with 1% bovine serum albumin (BSA) and incubated with the indicated antibody (Col1, 1:200, ABclonal, USA, Catalog No. A16891) overnight at 4 ℃, The cells were then stained with FITC-conjugated goat anti-rabbit secondary antibodies (1:200, ABclonal, USA, Catalog No. AS011) and subjected to 5 min of nuclear staining with DAPI. Images were acquired by confocal microscopy (InSIGHT Plus-IQ, Meridian, USA). The number of dots per cell was quantified, colocalization was performed in thresholded images, and masked images were used to calculate the results using ImageJ/NIS-elements BR 3.0 software.

For STIM1 staining, 10 μM TG or DMSO was used to treat cells for 10 min. The cells were fixed, blocked as mentioned above and then incubated with a primary antibody against STIM1 (1:200, ABclonal, USA, Catalog No. A7411) overnight at 4 ℃ before being stained with a Cy3 goat anti-rabbit secondary antibody (1:200, ABclonal, USA, Catalog No. AS007) and DAPI. Images were acquired by confocal microscopy (InSIGHT Plus-IQ, Meridian, USA). STIM1 redistribution was quantified as the ratio of fluorescence intensity in the peripheral region (F_p) to the fluorescence intensity of the total cell (F_{TOT}) by NIS-Elements BR 3.0 software.[4a]

For Calcein and Col1 staining, live cell were treated with Calcein-AM (10 μM, C001, Dojindo, Japan) for 30 min at 37 ℃. Then, the cells were fixed in 4% paraformaldehyde,

blocked with 1% bovine serum albumin (BSA), incubated with the indicated antibody (Col1, 1:200, ABclonal, USA, Catalog No. A16891) overnight at 4 ℃ and stained with a Cy3 goat anti-rabbit secondary antibody (1:200, ABclonal, USA, Catalog No. AS007) and DAPI. Images were acquired by confocal microscopy (InSIGHT Plus-IQ, Meridian, USA).

Cytosolic ($[Ca^{2+}]_{cyto}$) and ER ($[Ca^{2+}]_{ER}$) Ca^{2+} measurements: To visualize cytosolic Ca^{2+}, the Fura-2-AM fluorescent Ca^{2+} sensor (S1052, Beyotime, China) was used. After treated by drugs, cells were loaded with 5 μM Fura-2 for 30 min and stained with DAPI. Images were acquired by confocal microscopy (InSIGHT Plus-IQ, Meridian, USA) by alternating 340 nm and 380 nm wavelengths. NIS-elements software (Nikon) was used to process and analyze 340/380 ratiometric images. An increase in 340/380 ratio of 10% or more from baseline levels was considered a positive response to a ligand. Intensity was calculated based on ratiometric data.[11]

To evaluate Ca^{2+} changes in the cytoplasm after treatment, the Fluo-4/AM (S1060, Beyotime, China) fluorescent Ca^{2+} sensor was used according to previous study.[4] The Ca^{2+} free Hank's Balanced Salt Solution (HBSS) contained 140 mM NaCl, 5 mM KCl, 1 mM $MgCl_2$, 1 mM EGTA, 10 mM glucose, and 20 mM HEPES (pH = 7.3). Cells were incubated with 5 mM Fluo-4/AM for 40 min and washed twice with HBSS. ATP·2Na·3H_2O (100 μM, A1852, Sigma, USA) or thapsigargin (10 μM), Ion (10 μM) in Ca^{2+}-free HBSS or OM was used to stimulate Ca^{2+} release. Experiments were performed at 22 ℃. The data were obtained at interval of indicated seconds using MetaMorph (Molecular Devices, version 7.0). Images were obtained, background was subtracted and then ratio images were collected. The Ca^{2+}

signal was presented as the mean of the relative change in fluorescence intensity normalized to baseline intensity (*ΔF/F*). Trace graphs were generated in Excel and GraphPad Prism 7.0. Each trace line is an average of Ca^{2+} responses from all cells in each group. *ΔF/F* data are shown as the mean ± SEM. The area under the curve (AUC) was determined with GraphPad Prism 7.0.

$[Ca^{2+}]_{ER}$ detection with D1ER (pcDNA-D1ER was a gift from Amy Palmer & Roger Tsien (Addgene plasmid #36325; http://n2t.net/addgene:36325; RRID:Addgene_36325) was performed on an Olympus FV1000 confocal microscope as previously described.[12] Briefly, the $[Ca^{2+}]_{ER}$ change was calculated as $R-R_{min}$. R_{min} was obtained via 10 μM TG treatment followed by 10 μM ionomycin and 20 mM EGTA treatment. EGTA (5 mM) and HEDTA (5 mM) were used to buffer free $[Ca^{2+}]_{ER}$ below 100 μM, and HEDTA (10 mM) was used to buffer free $[Ca^{2+}]_{ER}$ between 100 μM and 1 mM (Max Chelator Winmaxc, version 2.51). $ATP{\cdot}2Na{\cdot}3H_2O$ (100 μM) or TG (10 μM) in Ca^{2+}-free HBSS was used to evoke ER Ca^{2+} release. CFP fluorophores were excited by means of a 435 nm filter. CFP and FRET images were captured by means of a CCD camera (C9100-13, Hamamatsu, Japan) at 535 nm (YFP emission) and 480 nm (CFP emission), respectively. The YFP and CFP intensities were corrected for background and the ratio R was defined as follows: $R = (YFP_{cell} - YFP_{background})(CFP_{cell} - CFP_{background})^{-1}$. All the images were background corrected and ratios were divided by the mean ratio recorded during the initial 2 minutes to minimize the variability between cells (R/Ro). Each trace line is an average of Ca^{2+} responses from all cells in each group. Data analysis was conducted in EXCEL and GraphPad Prism 7.0. and shown as mean ± SEM.

To chelate cytosolic Ca^{2+}, 40 μM BAPTA or an equivalent amount of DMSO was added

to the solution. The OM was refreshed with OM containing 8% ethanol to stimulate ER Ca^{2+} inflow and was then replaced with 100 μM ATP·2Na·3H_2O or 10 μM TG in Ca^{2+}-free HBSS. Data analysis was conducted in GraphPad Prism 7.0.

References

[1] a) C. Tang, Y. Wei, L. Gu, Q. Zhang, M. Li, G. Yuan, Y. He, L. Huang, Y. Liu, Y. Zhang, *Adv Sci (Weinh)* **2020**, 7, 1902536; b) J. Zhang, L. Tang, H. Qi, Q. Zhao, Y. Liu, Y. Zhang, *Adv Healthc Mater* **2019**, 8, e1901030.

[2] H. Zhu, Z. K. Guo, X. X. Jiang, H. Li, X. Y. Wang, H. Y. Yao, Y. Zhang, N. Mao, *Nat Protoc* **2010**, 5, 550.

[3] Y. Zhang, A. J. Edgley, A. J. Cox, A. K. Powell, B. Wang, A. R. Kompa, D. I. Stapleton, S. C. Zammit, S. J. Williams, H. Krum, R. E. Gilbert, D. J. Kelly, *Eur J Heart Fail* **2012**, 14, 549.

[4] a) J. Bi, W. Wang, Z. Liu, X. Huang, Q. Jiang, G. Liu, Y. Wang, X. Huang, *Cell Metab* **2014**, 19, 861; b) Q. C. Wang, Q. Zheng, H. Tan, B. Zhang, X. Li, Y. Yang, J. Yu, Y. Liu, H. Chai, X. Wang, Z. Sun, J. Q. Wang, S. Zhu, F. Wang, M. Yang, C. Guo, H. Wang, Q. Zheng, Y. Li, Q. Chen, A. Zhou, T. S. Tang, *Cell* **2016**, 165, 1454.

[5] a) L. Cinque, A. Forrester, R. Bartolomeo, M. Svelto, R. Venditti, S. Montefusco, E. Polishchuk, E. Nusco, A. Rossi, D. L. Medina, R. Polishchuk, M. A. De Matteis, C. Settembre, *Nature* **2015**, 528, 272; b) S. Stegen, K. Laperre, G. Eelen, G. Rinaldi, P. Fraisl, S. Torrekens, R. Van Looveren, S. Loopmans, G. Bultynck, S. Vinckier, F. Meersman, P. H. Maxwell, J. Rai, M. Weis, D. R. Eyre, B. Ghesquiere, S. M. Fendt, P. Carmeliet, G. Carmeliet, *Nature* **2019**, 565, 511.

[6] S. Boonrungsiman, E. Gentleman, R. Carzaniga, N. D. Evans, D. W. McComb, A. E. Porter, M.

M. Stevens, *Proc Natl Acad Sci U S A* **2012**, 109, 14170.

[7] D. D. Pei, J. L. Sun, C. H. Zhu, F. C. Tian, K. Jiao, M. R. Anderson, C. Yiu, C. Huang, C. X. Jin, B. E. Bergeron, J. H. Chen, F. R. Tay, L. N. Niu, *Adv Sci (Weinh)* **2018**, 5, 1800873.

[8] a) H. Kim, T. Kim, B. C. Jeong, I. T. Cho, D. Han, N. Takegahara, T. Negishi-Koga, H. Takayanagi, J. H. Lee, J. Y. Sul, V. Prasad, S. H. Lee, Y. Choi, *Cell Metab* **2013**, 17, 249; b) S. M. Gehrig, C. van der Poel, T. A. Sayer, J. D. Schertzer, D. C. Henstridge, J. E. Church, S. Lamon, A. P. Russell, K. E. Davies, M. A. Febbraio, G. S. Lynch, *Nature* **2012**, 484, 394.

[9] a) W. A. Cabral, M. Ishikawa, M. Garten, E. N. Makareeva, B. M. Sargent, M. Weis, A. M. Barnes, E. A. Webb, N. J. Shaw, L. Ala-Kokko, F. L. Lacbawan, W. Hogler, S. Leikin, P. S. Blank, J. Zimmerberg, D. R. Eyre, Y. Yamada, J. C. Marini, *PLoS Genet* **2016**, 12, e1006156; b) L. Van Duyn Graham, M. T. Sweetwyne, M. A. Pallero, J. E. Murphy-Ullrich, *J Biol Chem* **2010**, 285, 7067.

[10] a) S. Kuchay, C. Giorgi, D. Simoneschi, J. Pagan, S. Missiroli, A. Saraf, L. Florens, M. P. Washburn, A. Collazo-Lorduy, M. Castillo-Martin, C. Cordon-Cardo, S. M. Sebti, P. Pinton, M. Pagano, *Nature* **2017**, 546, 554; b) A. Carreras-Sureda, F. Jana, H. Urra, S. Durand, D. E. Mortenson, A. Sagredo, G. Bustos, Y. Hazari, E. Ramos-Fernandez, M. L. Sassano, P. Pihan, A. R. van Vliet, M. Gonzalez-Quiroz, A. K. Torres, C. Tapia-Rojas, M. Kerkhofs, R. Vicente, R. J. Kaufman, N. C. Inestrosa, C. Gonzalez-Billault, R. L. Wiseman, P. Agostinis, G. Bultynck, F. A. Court, G. Kroemer, J. C. Cardenas, C. Hetz, *Nat Cell Biol* **2019**, 21, 755.

[11] F. A. Pinho-Ribeiro, B. Baddal, R. Haarsma, M. O'Seaghdha, N. J. Yang, K. J. Blake, M. Portley, W. A. Verri, J. B. Dale, M. R. Wessels, I. M. Chiu, *Cell* **2018**, 173, 1083.

[12] a) A. E. Palmer, C. Jin, J. C. Reed, R. Y. Tsien, *Proc Natl Acad Sci U S A* **2004**, 101, 17404; b)

W. W. Shen, M. Frieden, N. Demaurex, *J Biol Chem* **2011**, 286, 36448.

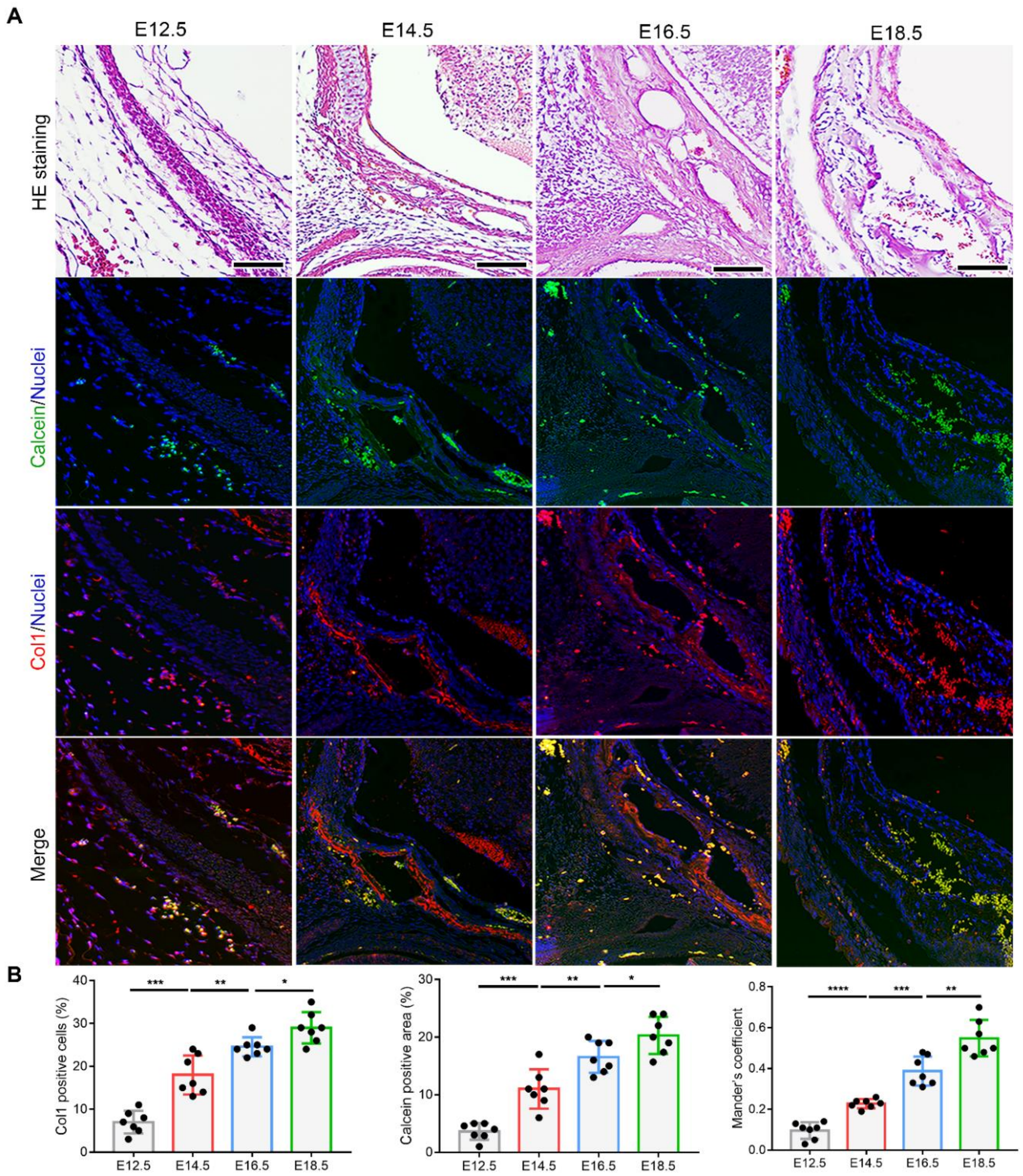

Figure S1. Histological analysis of cranium collected from different time points. A) Haematoxylin and eosin (HE) staining of mineralization front of cranium. Scale bar = 50 µm. Calcein-AM (Calcein) labeled Ca^{2+} deposition (green) and red fluorescence indicates the Col1 expression. Yellow suggests co-localization of fluorescent staining. B) Quantification analysis of images in A). Colocalization quantification is showed by Mander's coefficient. $N = 7$. * $P < 0.05$, ** $P < 0.005$, *** $P < 0.0005$, and **** $P < 0.0001$) All of the experiments are performed at least three times.

Unless otherwise stated, data presented in all supplementary figures are the mean standard deviation with one-way ANOVA with Tukey's post-test.

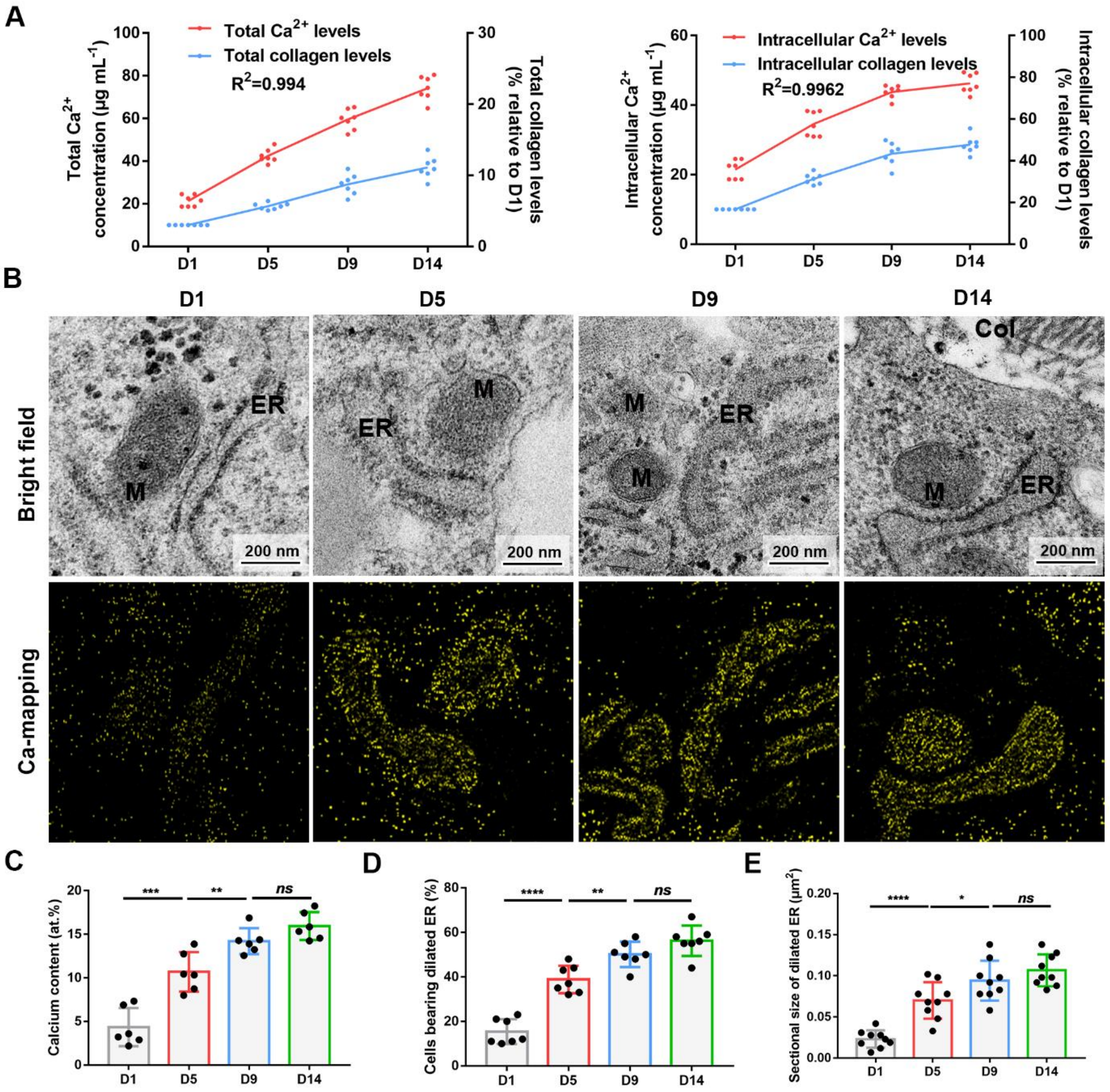

Figure S2. Correlation between Ca^{2+} and Col1 during mineralization *in vitro*. A) *Left panel;* Total Ca^{2+} concentrations and collagen levels of whole cell samples. N = 7; means of each group are connected; ** P = 0.003. *Right panel;* Intracellular Ca^{2+} concentrations and collagen levels. N = 7; means of each group are connected; ** P = 0.0019. B) STEM–EDX elemental mapping. Scale bar = 200 nm. M, mitochondria; N, nucleus; ER, endoplasmic reticulum. C) The comparison of elemental compositions (at %) among regions containing ER in the same area of the groups. N = 6. D) The comparison of the percentage of cells bearing dilated ER. Dilated ER elements are defined as ribosome-studded organelles with expanded lumina (> 0.05 μm^2 in cross-sectional area). N = 7. E) The comparison of sectional size of dilated ER. N = 9. * P < 0.05, ** P < 0.005, *** P < 0.0005, and **** P < 0.0001, *ns*, no significant difference. All of the experiments are performed at least three times.

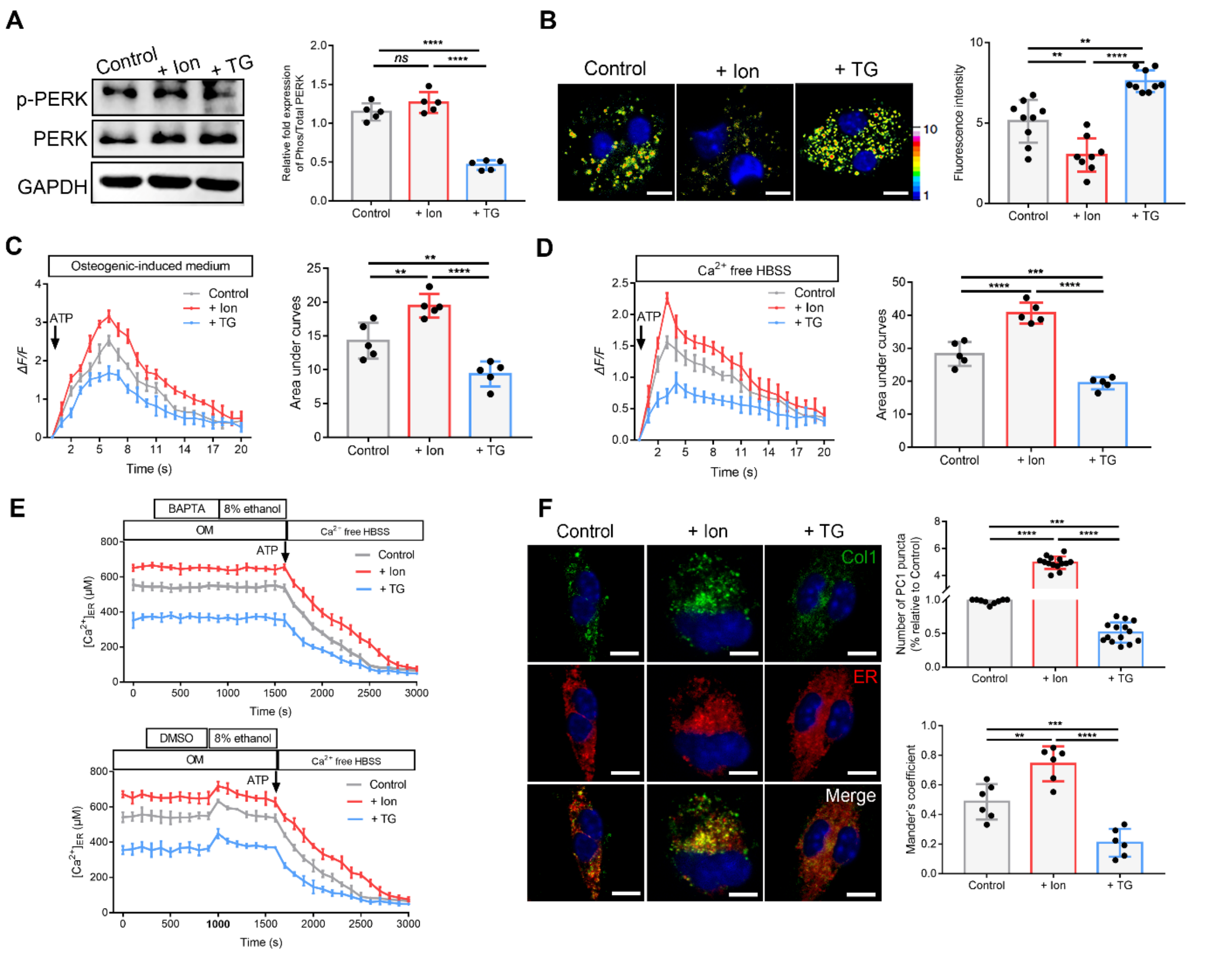

Figure S3. Characteristics of Ca^{2+} changes after treatments of Ion or TG. A) p-PERK, PERK and GAPDH (loading control) immunoblots. N = 5; relative phosphorylation of p-PERK was calculated relative to PERK; two-tailed Student's *t*-test. B) Fura-2-AM fluorescence changes. Scale bar = 10 μm. Histogram shows the fluorescence intensity (arbitrary units). N = 9. C-D) The cytosolic Ca^{2+} change tested by Fluo-4-AM (Fluo 4) (presented in $\Delta F/F$) with time after stimulation with 100 μM ATP in calcium-free solution or OM. Error bars represent ± SEM (C, control group, grey line, N = 50; + Ion group, red line, N = 45; + TG group, blue line, N = 50; D, control group, N = 50; + Ion group, N = 40; + TG group, N = 40). Bar charts show the area under the curves (AUC). N = 5. E) ER Ca^{2+} changes manipulated by 8% ethanol and ATP in 40 μM BAPTA-AM loaded cells. DMSO were used as control. Each trace line is shown in mean ± SEM (control group, grey line, N = 10; + Ion group, red line, N = 10; + TG group, blue line, N = 10). F) Representative confocal images of cells in three groups stained with Col1 (green), ER-tracker (red) and nuclei (blue). Scale bar = 10 μm. The graph shows percentage of cells with Col1 dots. N = 14. Colocalization quantification is showed by Mander's coefficient. N = 6. All of the experiments are performed at least three times. ** $P < 0.005$, *** $P < 0.0005$, and **** $P < 0.0001$.

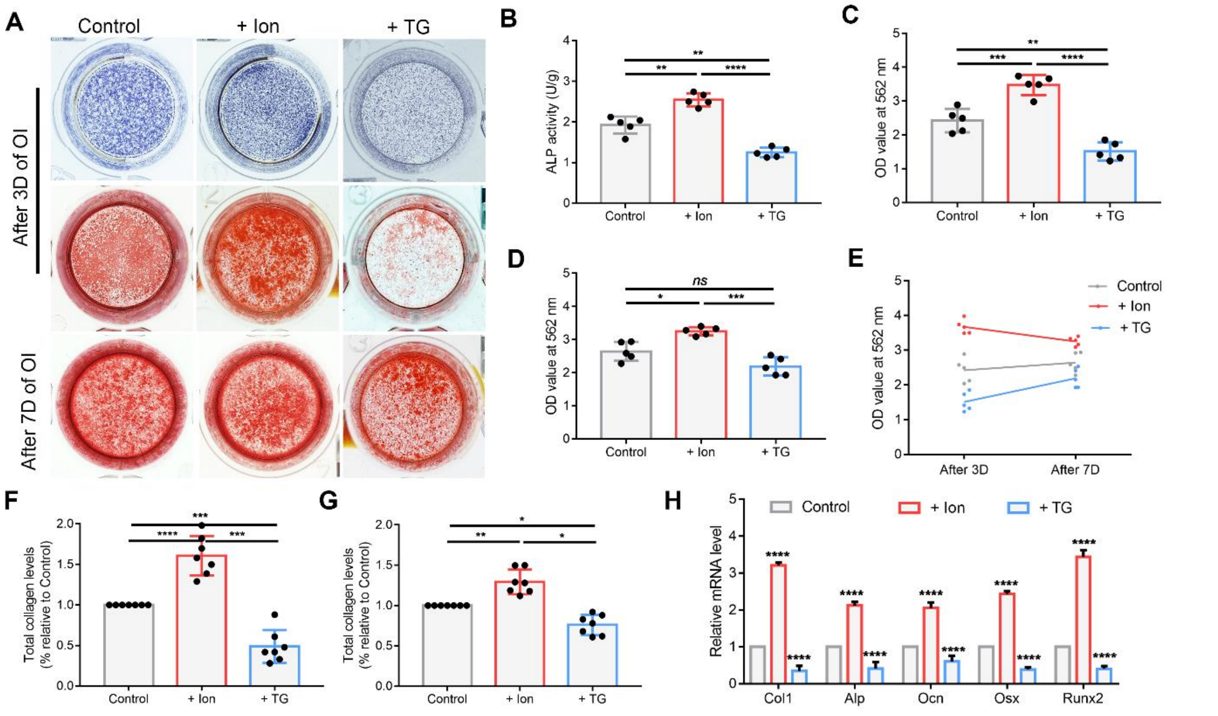

Figure S4. Effects of ER Ca^{2+} changes on mineralization. A) ALP and ARS staining. Drugs are added into OM after 3 or 7 days of induction for 6 h, respectively. ALP staining is performed at induction day 7th. The ARS staining is uniformly performed at induction day 14th. B) ALP activity. $N = 5$. C) Semi-quantification of the second row of (A). $N = 5$. D) Semi-quantification of the third row of A). $N = 5$. E) Changes of OD value in *C*, *D*). Means of each group are connected. $N = 5$. F-G) Total collagen levels detected at induction day 14th of second and third row in A). $N = 7$; two-tailed Student's *t*-test. H) mRNA levels of key osteogenic markers in three groups. $N = 6$. two-way ANOVA with Tukey's post-test. All of the experiments are performed at least three times. $*P < 0.05$, $**P < 0.005$, $***P < 0.0005$, and $****P < 0.0001$.

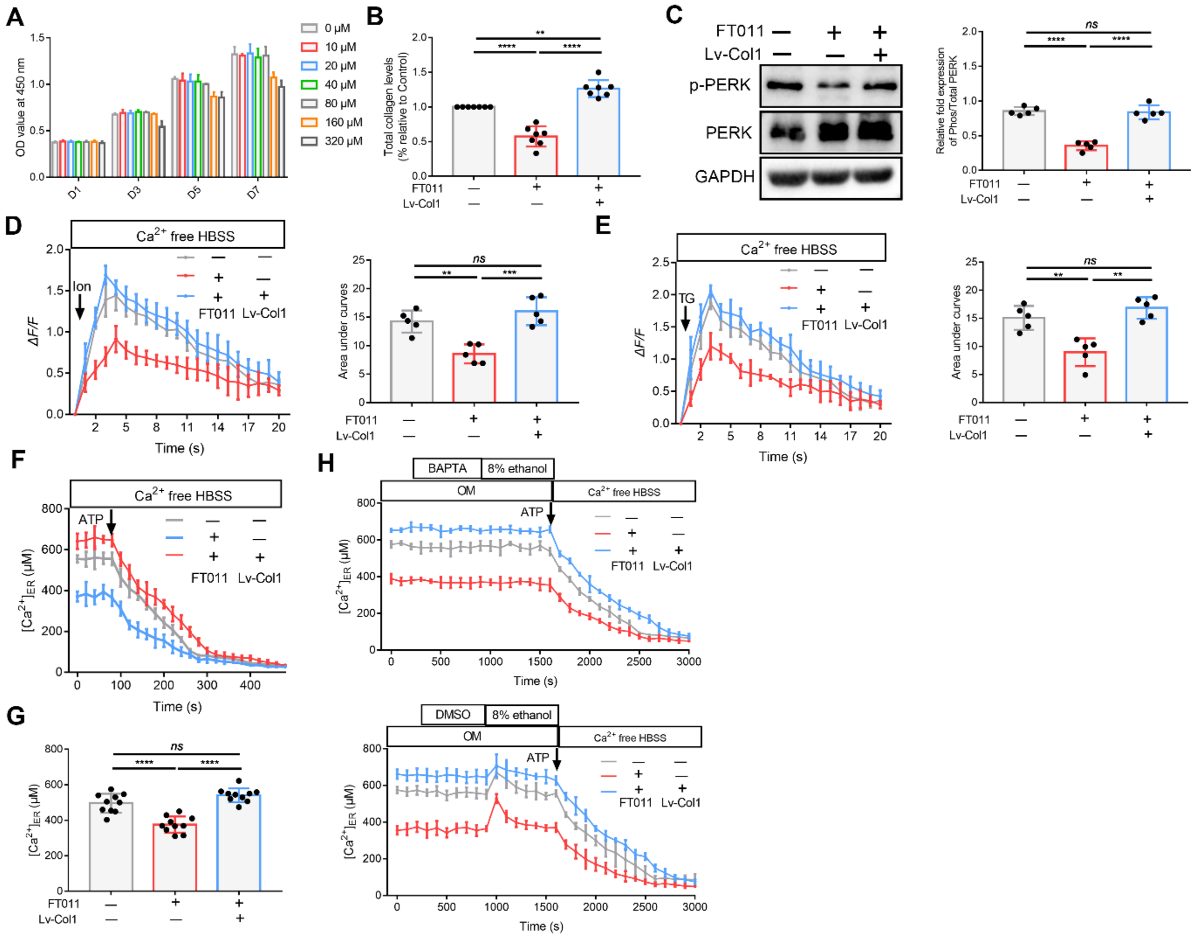

Figure S5. Characteristics of Ca^{2+} concentrations after collagen inhibition and retrieval. A) CCK8 assay. *N* = 8. B) Inhibition and retrieval effect of collagen by the treatment of 80 μM FT011 and highest dose of Lv-Col1. *N* = 7; two-tailed Student's *t*-test. C) p-PERK, PERK and GAPDH (loading control) immunoblots. *N* = 5; relative phosphorylation of p-PERK was calculated with gray value and analyzed using two-tailed Student's *t*-test. D-E) The cytosolic Ca^{2+} change tested by Fluo-4-AM (Fluo 4) (*ΔF/F*) with time after stimulation with 10 μM Ion or 10 μM TG in calcium-free solution. Error bars represent ± SEM (D, control group, grey line, *N* = 30; FT011 group, red line, *N* = 25; retrieval group, blue line, *N* = 30; E, control group, *N* = 30; FT011 group, *N* = 25; retrieval group, *N* = 30). Bar chart showing the area under the curves (AUC). *N* = 5. F) ER Ca^{2+} levels were monitored by D1ER in control (grey line, *N* = 30), FT011 group (red line, *N* = 35), retrieval group (blue line, *N* = 30) after the stimulation of 100 μM ATP. Error bars represent ± SEM. G) Histogram shows the average ER Ca^{2+} levels in resting cells. *N* = 10. H) ER Ca^{2+} changes manipulated by 8% ethanol and ATP in 40 μM BAPTA-AM loaded cells. DMSO were used as control. Each trace line is shown in mean ± SEM (control group, grey line, *N* = 10; + Ion group, red line, *N* = 10; + TG group, blue line, *N* = 10). All of the experiments are performed at least three times. *P < 0.05, **P < 0.005, ***P < 0.0005, and ****P < 0.0001, *ns*, no significant difference.

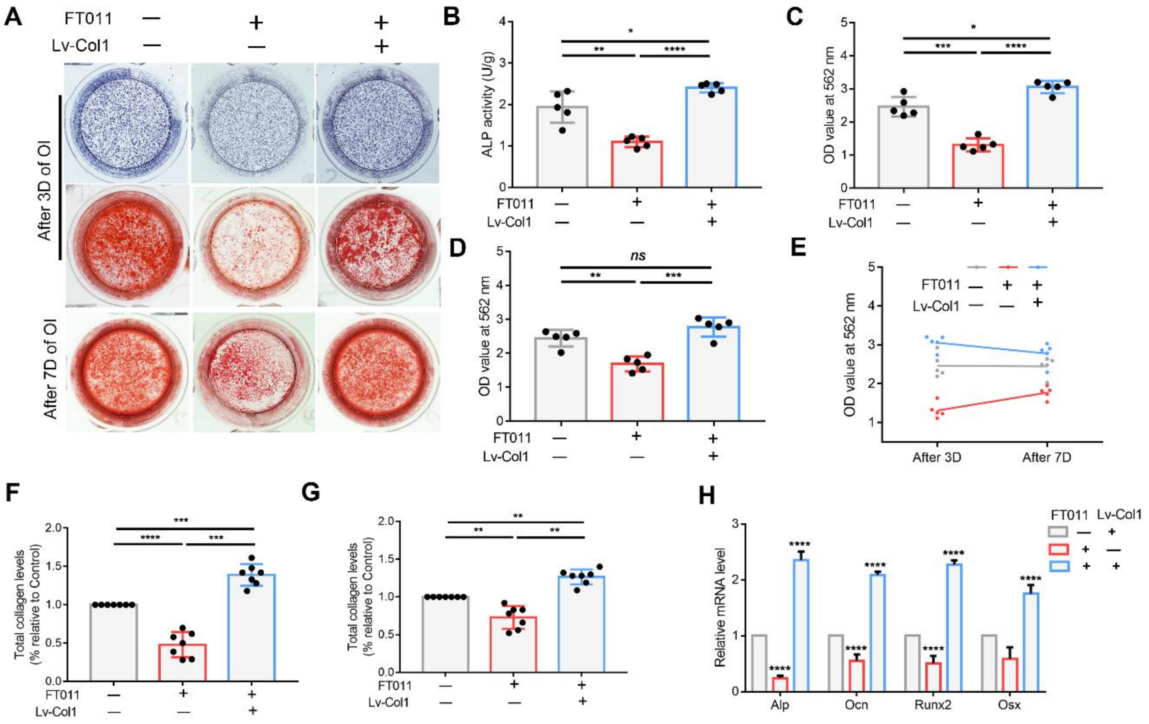

Figure S6. Effects of collagen expression changes on mineralization. A) ALP and ARS staining. FT011 is added into OM after 3 or 7 days of induction for 2h, then with or without retrieval, respectively. ALP staining is performed at induction day 7th. The ARS staining is uniformly performed at induction day 14th. B) ALP activity. $N = 5$. C) Semi-quantification of the second row of A). $N = 5$. D) Semi-quantification of the third row of A). $N = 5$. E) Changes of OD value in C, D). Means of each group are connected. $N = 5$. F-G) Total collagen levels detected at induction day 14th of second and third row in A). $N = 7$; two-tailed Student's *t*-test. H) mRNA levels of key osteogenic markers in three groups. $N = 6$; two-way ANOVA with Tukey's post-test. All of the experiments are performed at least three times. $*P < 0.05$, $**P < 0.005$, $***P < 0.0005$, and $****P < 0.0001$.

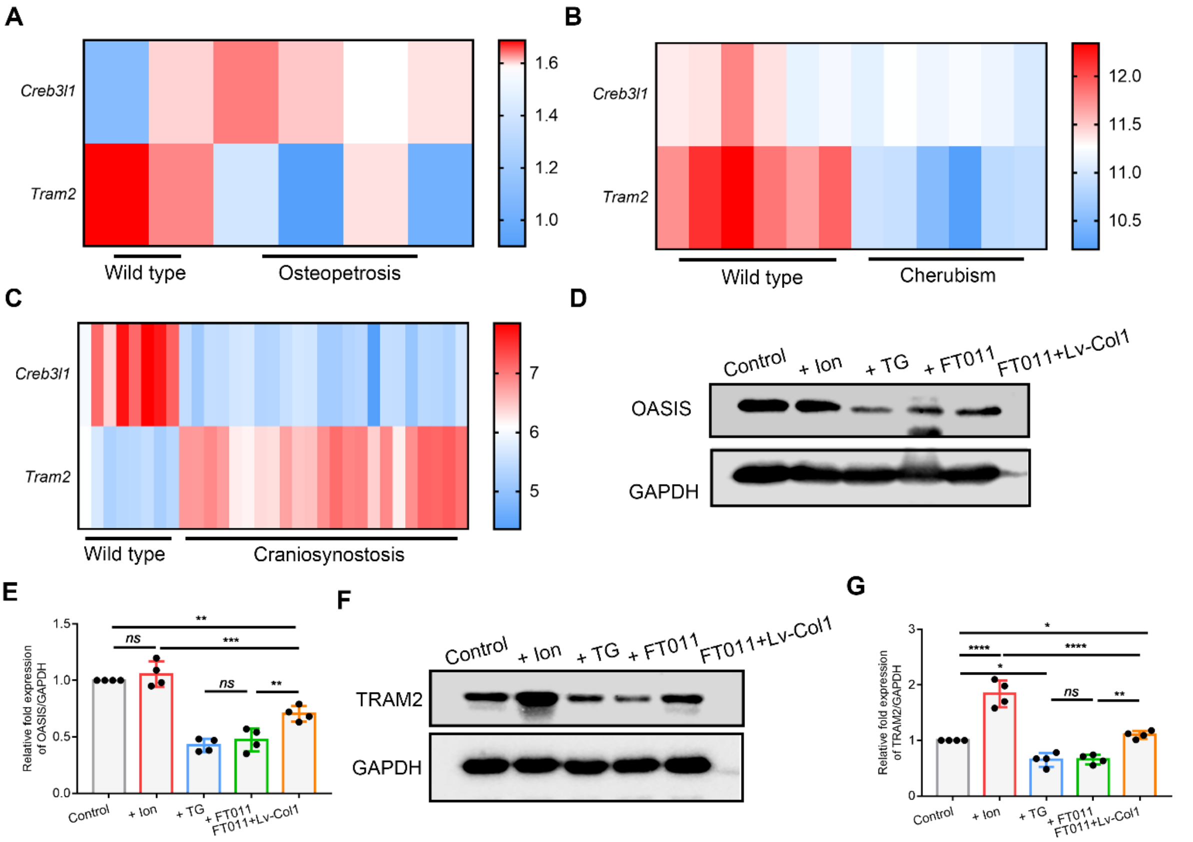

Figure S7. Preliminary determination of TRAM2 as candidate. A-C) Heatmaps of 2 genes differently expressed in three disease models. D) OASIS and GAPDH (loading control) immuneblots of five groups. E) Quantification of data in D). $N = 4$; two-tailed Student's *t*-test. F) TRAM2 and GAPDH (loading control) immunoblots of five groups. G) Quantification of data in F). $N = 4$; two-tailed Student's *t*-test. All of the experiments are performed at least three times. $^{*}P < 0.05$, $^{**}P < 0.005$, $^{***}P < 0.0005$, and $^{****}P < 0.0001$, *ns*, no significant difference.

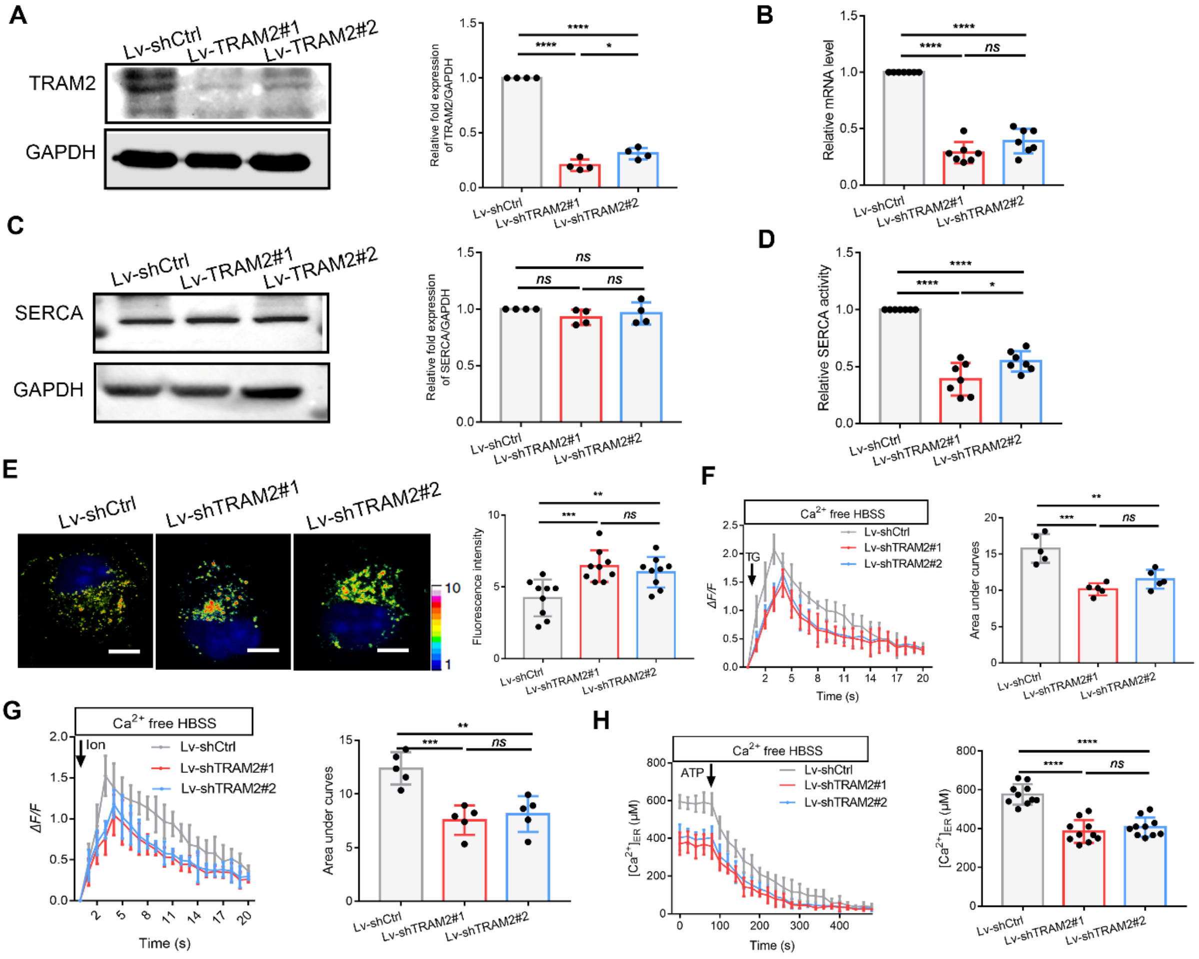

Figure S8. Ca^{2+} characteristics of TRAM2-knockdown cells. A) The knockdown efficiency of two shRNA. TRAM2 and GAPDH (loading control) immunoblots of three groups. Quantification of data. N = 4. B) Relative mRNA level of *Tram2* in cells with or without transfecting by shRNA. N = 7. C) SERCA2b and GAPDH (loading control) immunoblots of three groups. Quantification of data. N = 4. D) Relative SERCA2b activity in three groups. N = 7. E) Fura-2-AM fluorescence changes. Scale bar = 10 μm. Histogram shows the fluorescence intensity (arbitrary units). N = 9. F-G) Cytosolic Ca^{2+} changes in cells of three groups tested by Fluo4 ($\Delta F/F$) with time treated with 10 μM TG or 10 μM Ion in the absence of Ca^{2+}. Error bars represent ± SEM (control group, grey line, N = 50; knockdown group 1, red line, N = 35; knockdown group 2, blue line, N = 40). Bar chart showing the area under the curves (AUC). N = 5. H) ER Ca^{2+} levels were monitored by D1ER after the stimulation of 100 μM ATP. Control group, grey line, N = 40; knockdown group 1, red line, N = 45; knockdown group 2, blue line, N = 40. Error bars represent ± SEM. Histogram shows the average ER Ca^{2+} levels in resting cells in each group. N = 10. A-D) two-tailed Student's *t*-test. All of the experiments are performed at least three times. *P < 0.05, **P < 0.005, ***P < 0.0005, and ****P < 0.0001, *ns*, no significant difference.

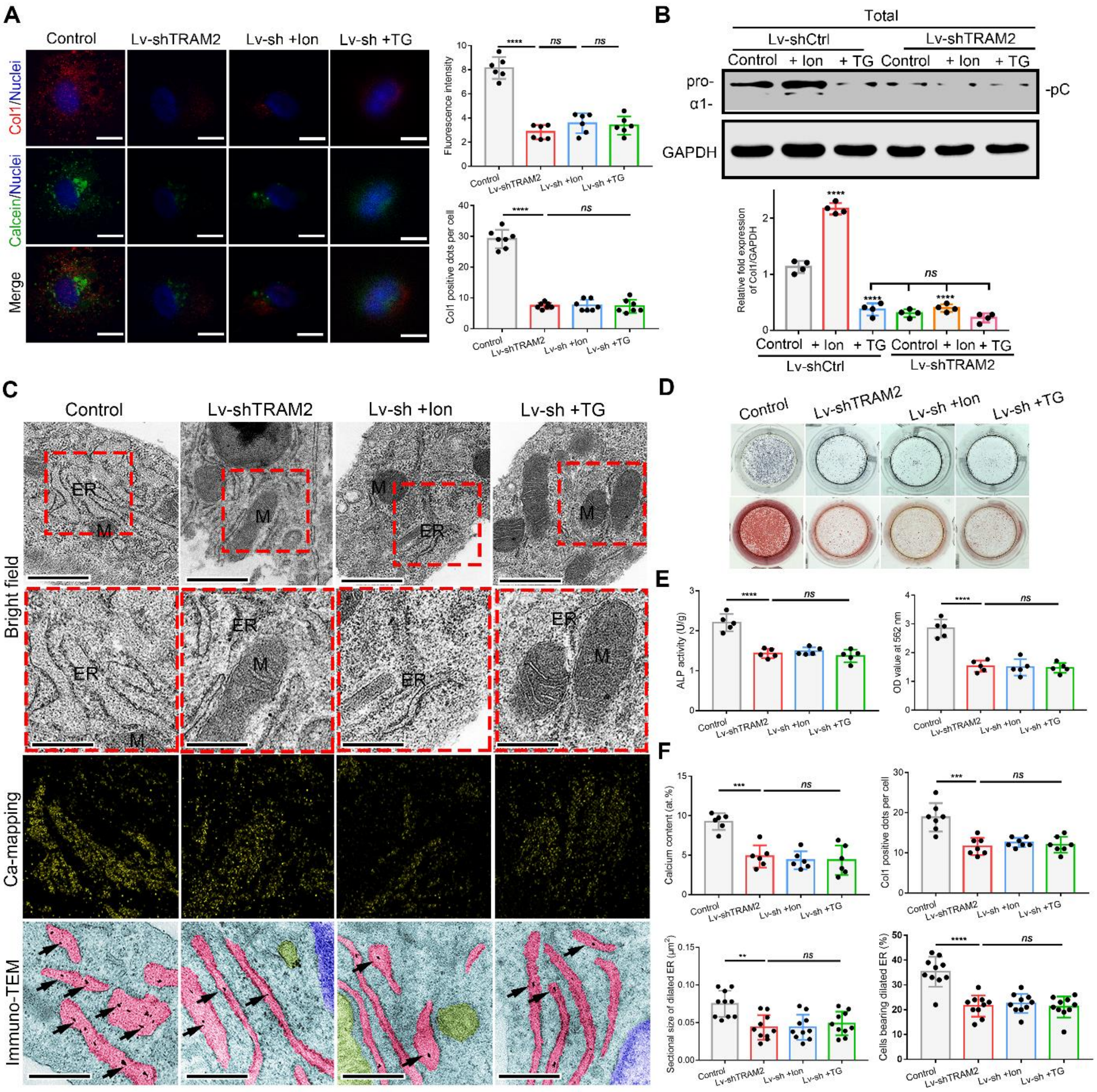
A
Control
Lv-shTRAM2
Lv-sh +Ion
Lv-sh +TG
Col1/Nuclei
Calcein/Nuclei
Merge
Fluorescence intensity
Col1 positive dots per cell
****
ns
B
Total
Lv-shCtrl
Lv-shTRAM2
Control
+ Ion
+ TG
pro-
α1-
-pC
GAPDH
Relative fold expression of Col1/GAPDH
C
Bright field
Ca-mapping
Immuno-TEM
ER
M
D
E
ALP activity (U/g)
OD value at 562 nm
F
Calcium content (at.%)
Col1 positive dots per cell
Sectional size of dilated ER (μm²)
Cells bearing dilated ER (%)
***
**

Figure S9. The useless effects of Ion and TG on TRAM2-knockdown cells. A) Representative confocal images of cells stained with Calcein (green), Col1 (red) and nuclei (blue). Scale bar = 10 μm. Quantification of data. The histogram shows fluorescence intensity (arbitrary units) and col1 positive dots per cell. $N = 9$. B) Col1 and GAPDH (loading control) immunoblots of total proteins. Pro is the unprocessed form with the N- and C-propeptides; pC is collagen with the N-propeptide cleaved; an α1(I) is the fully processed α(I) band. Quantification of data. $N = 4$; two-tailed Student's *t*-test. C) STEM-EDX elemental mapping and immune-TEM of three groups. Insets show a high magnification of selected areas. Scale bar = 500 nm and = 200 nm in insets. M, mitochondria; ER, endoplasmic reticulum. Unstained sections are pseudo-colored. ERs are pink. Arrows indicate the location of Col1. D) ALP and ARS staining of the mineralization of BMMSCs. E) ALP activity. $N = 5$. and semi-quantification of the ARS. $N = 5$. F) The comparison of elemental compositions (at %) among regions containing ER in the same area of the groups. $N = 6$. The comparison of Col1 positive dots per cell. $N = 7$. The comparison of sectional size of dilated ER. $N = 10$. And the comparison of the percentage of cells bearing dilated ER. $N = 10$. All of the experiments are performed at least three times. $^{*}P < 0.05$, $^{**}P < 0.005$, $^{***}P < 0.0005$, and $^{****}P < 0.0001$, *ns*, no significant difference. Lv-sh in this figure indicates Lv-shTRAM2.

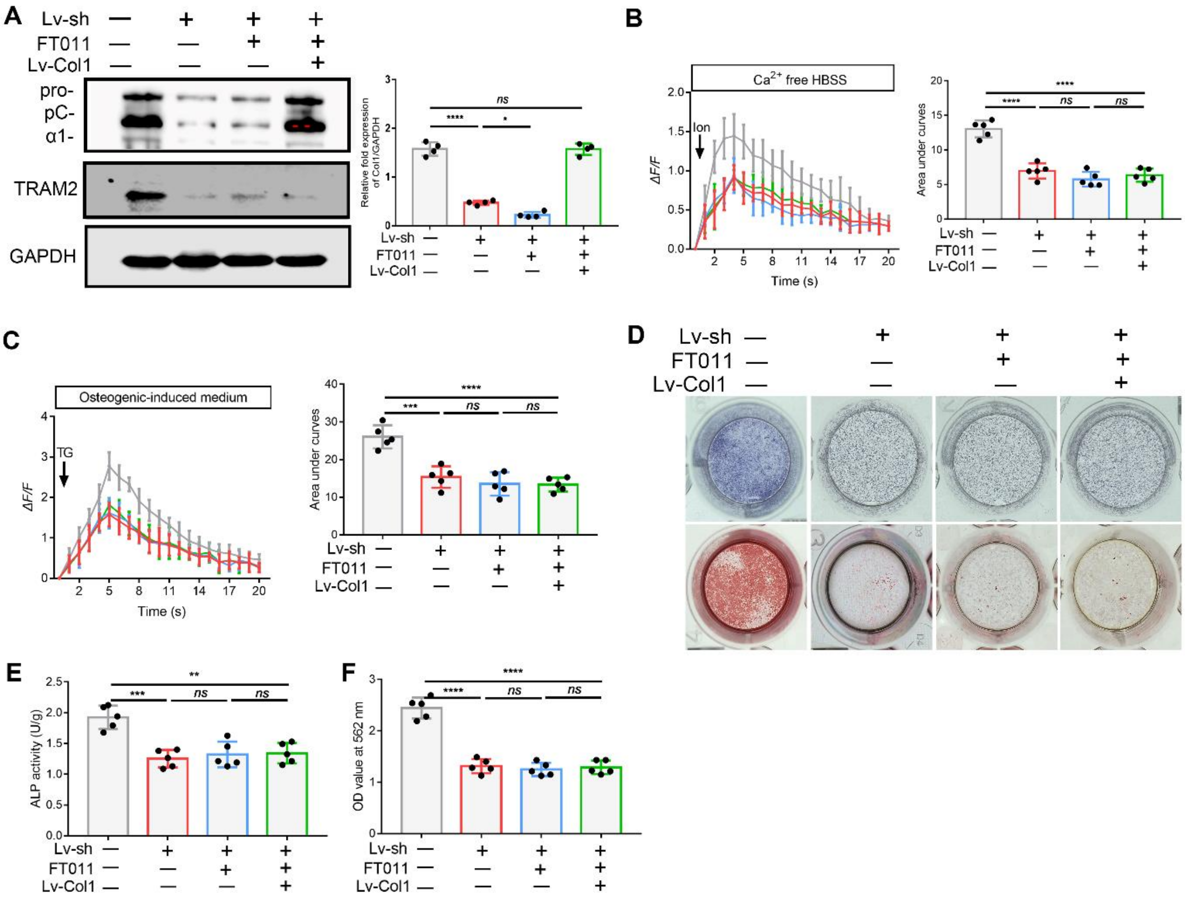

Figure S10. The effects of Col1 expression changes on TRAM2-knockdown cells. A) Col1, TRAM2 and GAPDH (loading control) immunoblots of total proteins. Quantification of data. N = 4; two-tailed Student's t-test. B-C) Cytosolic Ca^{2+} changes in cells tested by Fluo-4-AM (Fluo 4) (presented in $\Delta F/F$) with time treated with 10 μM TG or 10 μM Ion in the absence or presence of OM. Error bars represent ± SEM (control group, grey line, N = 50; knockdown group, red line, N = 35; + FT011, blue trace line, N = 40; retrieval group, green line, N = 45). Bar chart showing the area under the curves (AUC). N = 5. D) ALP and ARS staining. E) ALP activity in D). N = 5. F) Semi-quantification of the ARS in D). N = 5. All of the experiments are performed at least three times. *P < 0.05, **P < 0.005, ***P < 0.0005, and ****P < 0.0001, ns, no significant difference. Lv-sh indicates Lv-shTRAM2.

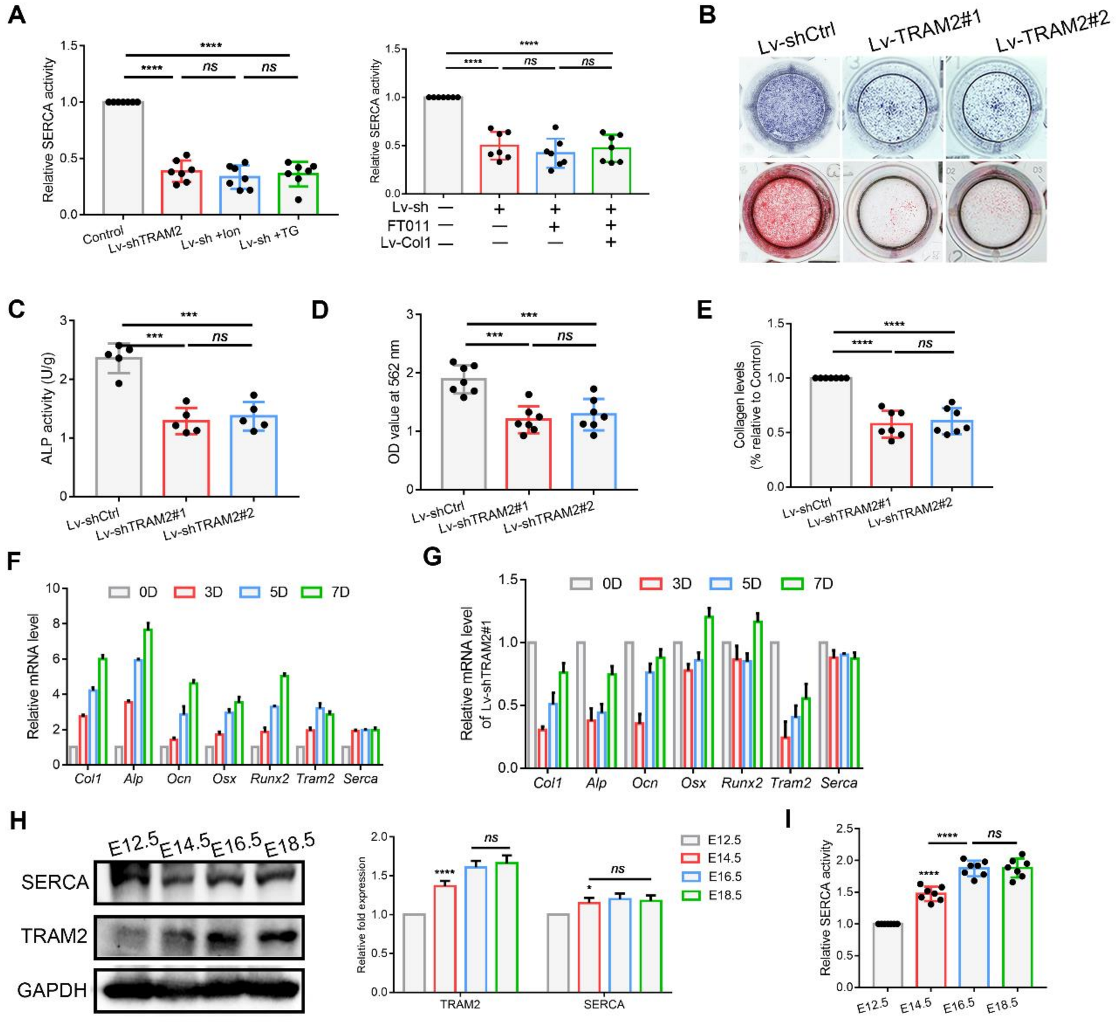

Figure S11. The importance of TRAM2 in osteogenesis. A) Relative SERCA2b activity. N = 7; two-tailed Student's t-test. B) ALP and ARS staining of the mineralization of normal or TRAM2 knockdown BMSCs. C-D) ALP activity. N = 5. And semi-quantification of the ARS. N = 5. E) Total collagen levels detected at induction day 14th in D). N = 7; two-tailed Student's t-test. F-G) mRNA levels of key osteogenic markers with or without TRAM2 knockdown. H) SERCA2b, TRAM2 and GAPDH (loading control) immunoblots of total proteins collected at several timepoints during cranial mineralization. Quantification of data. N = 4; two-tailed Student's t-test. I) Relative SERCA2b activity at several timepoints during cranial mineralization. N = 7; two-tailed Student's t-test. All of the experiments are performed at least three times. *P < 0.05, **P < 0.005, ***P < 0.0005, and ****P < 0.0001, *ns*, no significant difference. Lv-sh in this figure indicates Lv-shTRAM2.

Table S1. The shRNA sequences used in study.

Gene	Forward sequence	Reverse sequence
shTRAM2#1	ggatccGACAGTGCACTACCACTATGGctcgagccatagtggtagtgcactgtcTTTTTGAATTC	cctaggCTGTCACGTGATGGTGATACCgagctcggtatcaccatcacgtgacagAAAAACTTAAG
shTRAM2#2	ggatccGCTTCTATGTGATCGTAACAGctcgagctgttacgatcacatagaagcTTTTTGAATTC	cctaggCGAAGATACACTAGCATTGTCgagctcgacaatgctagtgtatcttcgAAAAACTTAAG

Table S2. The primer sequences used in study.

Gene	Forward primer sequence	Reverse primer sequence
Runx2	CCCAGTATGAGAGTAGGTGTCC	TGAAGGAGTGATTTGCCTTGT
Alp	CAACGAGGTCATCTCCGTGATG	TACCAGTTGCGGTTCACCGTGT
Col1	GAGCTGGTGTAATGGGTCCT	GAGACCCAGGAAGACCTCTG
Osx	GAGTAGGATTGTAGGATTGG	TGCTTGAGGAAGTTCACTATG
Ocn	AAGCAGGAGGGCAATAAGGT	TTTGTAGGCGGTCTTCAAGC
Tram2	TTCCGTAGGAGGACGAAAAGT	TAAGCCCGATGAGGACGCA
Serca2b	ACCTTTGCCGCTCATTTTCCAG	AGGCTGCACACACTCTTTACC
Gapdh	GCACCGTCAAGGCTGAGAAC	TGGTGAAGACGCCAGTGGA
